# Cyclin-Dependent Kinase-Like Function Is Shared by the Beta- and Gamma- Subset of the Conserved Herpesvirus Protein Kinases

**DOI:** 10.1371/journal.ppat.1001092

**Published:** 2010-09-09

**Authors:** Chad V. Kuny, Karen Chinchilla, Michael R. Culbertson, Robert F. Kalejta

**Affiliations:** 1 Institute for Molecular Virology and McArdle Laboratory for Cancer Research, University of Wisconsin-Madison, Madison, Wisconsin, United States of America; 2 Laboratories of Genetics and Molecular Biology, University of Wisconsin-Madison, Madison, Wisconsin, United States of America; Oregon Health & Science University, United States of America

## Abstract

The UL97 protein of human cytomegalovirus (HCMV, or HHV-5 (human herpesvirus 5)), is a kinase that phosphorylates the cellular retinoblastoma (Rb) tumor suppressor and lamin A/C proteins that are also substrates of cellular cyclin-dependent kinases (Cdks). A functional complementation assay has further shown that UL97 has authentic Cdk-like activity. The other seven human herpesviruses each encode a kinase with sequence and positional homology to UL97. These UL97-homologous proteins have been termed the conserved herpesvirus protein kinases (CHPKs) to distinguish them from other human herpesvirus-encoded kinases. To determine if the Cdk-like activities of UL97 were shared by all of the CHPKs, we individually expressed epitope-tagged alleles of each protein in human Saos-2 cells to test for Rb phosphorylation, human U-2 OS cells to monitor nuclear lamina disruption and lamin A phosphorylation, or *S. cerevisiae cdc28-13* mutant cells to directly assay for Cdk function. We found that the ability to phosphorylate Rb and lamin A, and to disrupt the nuclear lamina, was shared by all CHPKs from the beta- and gamma-herpesvirus families, but not by their alpha-herpesvirus homologs. Similarly, all but one of the beta and gamma CHPKs displayed *bona fide* Cdk activity in *S. cerevisiae*, while the alpha proteins did not. Thus, we have identified novel virally-encoded Cdk-like kinases, a nomenclature we abbreviate as v-Cdks. Interestingly, we found that other, non-Cdk-related activities reported for UL97 (dispersion of promyelocytic leukemia protein nuclear bodies (PML-NBs) and disruption of cytoplasmic or nuclear aggresomes) showed weak conservation among the CHPKs that, in general, did not segregate to specific viral families. Therefore, the genomic and evolutionary conservation of these kinases has not been fully maintained at the functional level. Our data indicate that these related kinases, some of which are targets of approved or developmental antiviral drugs, are likely to serve both overlapping and non-overlapping functions during viral infections.

## Author Summary

The eight human herpesviruses are ubiquitous pathogens associated with a wide spectrum of disease, and are grouped into three families (alpha-, beta-, and gamma-) based on sequence homology and tissue tropism. They encode sixteen kinase proteins that are grouped into three recognized families. Eight of these kinases, one from each virus, are found at homologous positions in their respective viral genomes and display limited but discernable amino acid similarity. These proteins have been designated the CHPKs for conserved herpesvirus-encoded protein kinases. We found that the beta and gamma CHPKs, but not the alpha CHPKs, share at least a subset of activities with the cellular cyclin-dependent kinases (Cdks) that are key components of cell cycle regulation. Furthermore, we discovered that the CHPKs, although evolutionarily-conserved, showed a somewhat surprising non-conservation of biological activities not associated with cellular Cdks. Knowledge of functional as well as sequence and positional conservation is essential for understanding the evolutionary relationships among viral, and between viral and cellular, kinases. Importantly, because viral kinases are the targets of approved and emerging antiviral treatments, cataloging both the common and unique activities of these proteins may help predict or explain successes or failures during antiviral drug development.

## Introduction

Kinases catalyze the transfer of phosphate groups onto targeted substrates. These phosphorylation events are fundamental to proper cellular function and viability and help control enzyme activity, signaling cascades, and protein trafficking, as well as a multitude of other pathways and processes [1]. The enzymatic activity of many kinases can be inhibited both potently and specifically with small molecules, making them suitable drug targets for clinical applications. In fact, kinases are now targets for lung and breast cancer chemotherapies [2], and importantly, for herpesviral infections as well [3], [4].

The human herpesviruses (HHVs) are large, enveloped viruses with double-stranded DNA genomes. Based on sequence homology and cellular tropisms, they are divided into three families, alpha, beta, and gamma. The alpha-herpesviruses are Herpes Simplex Virus type 1 (HSV-1, HHV-1), type 2 (HSV-2, HHV-2), and Varicella Zoster Virus (VZV, HHV-3). These viruses complete productive (lytic) replication cycles in epithelial cells, and establish life-long latency in sensory neurons. They cause recurrent and sometimes painful skin lesions and, in rare cases, meningitis [5], [6]. The beta-herpesviruses are Human Cytomegalovirus (HCMV, HHV-5), and the roseolaviruses Human Herpesviruses 6A and 6B (HHV-6), and Human Herpesvirus 7 (HHV-7). These viruses are classified as lymphotropic because lymphocytes likely serve as a latent reservoir, but they replicate productively in many cell types. Beta-herpesviruses cause severe disease in patients with immature or compromised immune function, and may exacerbate certain chronic ailments in otherwise healthy patients [7], [8]. The gamma-herpesviruses are Epstein-Barr Virus (EBV, HHV-4) and Kaposi's Sarcoma Associated Herpesvirus (KSHV, HHV-8). These viruses are also characterized by a lymphocyte tropism, but can also infect epithelial and endothelial cells. They are causally associated with human cancers [9], [10].

As obligate intracellular parasites, viruses must manipulate cellular processes to facilitate their own replication. Because protein phosphorylation events are so crucial to cellular activity, it is not surprising that they are key targets of regulation during viral infections. While many (perhaps all) families of viruses manipulate the activity of cellular kinases, only two families, the poxviruses [11], [12] and the herpesviruses [13], [14], [15] encode viral proteins with confirmed kinase activity. However, rotavirus, a member of the *Reoviridae* family of double stranded RNA viruses, encodes a protein called NSP5 that may be a functional kinase [16].

At least eighteen human herpesvirus proteins are reported to possess protein kinase activity. Sixteen of these are grouped into three distinct families, (Us3, UL13 and thymidine kinase) based on amino acid sequence homology (Table 1). The other two human herpesvirus proteins reported to have kinase activity are HHV-2 ICP10 [17] and HHV-5 pp65 [18]. These proteins do not appear to be members of any known viral kinase family. Among the conserved kinase families, only alpha-herpesviruses encode the Us3 family of kinases [14] that, among other functions, prevent apoptosis [19], [20] and disrupt the nuclear lamina [21], [22], [23]. Both the alpha- and gamma-herpesviruses encode thymidine kinase family members that, as their name implies, phosphorylate nucleosides, including thymidine [24]. Importantly, viral thymidine kinases can also phosphorylate unnatural nucleoside analogs (such as ganciclovir and its derivatives) that act as chain terminators for viral DNA replication, and constitute an important subset of anti-herpesviral drugs [24]. The third family of herpesviral kinases was originally termed the UL13 family [25], [26]. However, because these represent the only homologous kinases (at the level of genome position and amino acid sequence) found in every human herpesvirus, they were renamed the CHPKs for conserved herpesvirus-encoded protein kinases [13], [27]. The individual members of the human CHPKs are named UL13 (HHV-1 and -2), ORF47 (HHV-3), BGLF4 (HHV-4), UL97 (HHV-5), U69 (HHV-6 and -7), and ORF36 (HHV-8) (Table 2).

**Table 1:** Kinases encoded by the human herpesviruses.

Virus (HHV)	UL13 Family	Us3 Family	Thymidine Kinase
**HSV-1 (1)**	UL13	Us3	UL23
**HSV-2 (2)**	UL13	Us3	UL23
**VZV (3)**	ORF47	ORF66	ORF36
**EBV (4)**	BGLF4		BXLF1
**HCMV (5)**	UL97		
**HHV-6 (6)**	U69		
**HHV-7 (7)**	U69		
**KSHV (8)**	ORF36		ORF21

Human herpesvirus-encoded (HHV) kinases are grouped into families based on their sequence and positional homology. The commonly utilized gene or protein name is listed.

**Table 2:** CHPK alleles used in this study.

HHV number	Common name	Virus strain	CHPK name	Predicted MW	Accession number	KD mutation
**1**	HSV-1	KOS	UL13	57 kDa	HM584913	N/A
**2**	HSV-2	186	UL13	57 kDa	NC_001798	N/A
**3**	VZV	VZV32	ORF47	57 kDa	AF314218.1	N/A
**4**	EBV	B95-8	BGLF4	48 kDa	DQ279927	K102I
**5**	HCMV	AD169	UL97	78 kDa	X17403	K355M
**6**	HHV-6	U1102	U69	64 kDa	X83413.1	K218G
**7**	HHV-7	RK	U69	63 kDa	NC_001716	K202N
**8**	KSHV	GK18	ORF36	50 kDa	AF148805	K108Q

The indicated viral strains (delineated by the human herpesvirus number 1–8 as well as the commonly-used virus name) served as templates for PCR reactions generating the CHPK alleles analyzed in this study. Accession numbers indicate the GenBank entries used for sequence confirmation. The amino acid substitutions in the kinase-deficient (KD) mutant alleles are indicated.

The CHPKs are not absolutely required for viral replication in cell culture, but deletion mutants are severely attenuated for viral growth [28], [29], [30], [31], [32]. They are expressed with early-late kinetics and are incorporated into virions [33], [34], [35], [36], [37]. Demonstrated or postulated functions for the CHPKs during viral replication include tegument disassembly [38], [39], modulation of gene expression [28], [31], [40], stimulation of viral DNA replication [31], [41], [42], [43], and facilitating capsid nuclear egress in part through disruption of the nuclear lamina [31], [44], [45]. Recently, the CHPK encoded by the UL97 gene of HHV-5 (HCMV) was shown to directly phosphorylate the cellular retinoblastoma (Rb) tumor suppressor protein both *in vivo* and *in vitro*, on residues that are normally targeted by the cellular cyclin-dependent kinase (Cdk) proteins that control cell cycle progression [46], [47]. UL97 was also found to phosphorylate lamin A/C proteins *in vitro* on Cdk phosphorylation sites [45], and to rescue the G1-to-S cell cycle defect of *Saccharomyces cerevisiae* cells lacking Cdk function. Significantly, this yeast complementation assay demonstrated that UL97 can functionally substitute for cellular Cdks [46], indicating that the kinase has Cdk activity, and marking UL97 as the first identified v-Cdk, an abbreviation for the term virally-encoded Cdk-like kinase.

Here we show that the CHPKs encoded by the beta- and gamma-herpesviruses are all capable of inducing Rb phosphorylation *in vivo* on residues that inactivate the cell cycle inhibitory and tumor suppressor function of this protein. They can also induce lamin A phosphorylation and disrupt the nuclear lamina. Importantly, all the beta- and gamma-herpesvirus CHPKs, with the exception of the HHV-8 (KSHV) ORF36 protein, displayed authentic Cdk function in the yeast complementation assay. The alphaherpesvirus CHPKs were unable to phosphorylate Rb or lamin A, efficiently disrupt the nuclear lamina, or act as Cdks in *S. cerevisiae*. When we assayed all eight kinases for additional non-Cdk functions against cellular proteins that were previously reported for UL97, we found that despite the evolutionary conservation of these proteins, functional conservation was poor. Altogether, our study identifies a subset of the CHPKs as viral Cdk-like kinases (v-Cdks), but also indicates that the positional homology and amino acid similarity of these protein kinases does not always translate into common substrates or similar biological activities for these proteins.

## Results

### Analysis of the steady state levels and sub-cellular localization of ectopically-expressed, epitope-tagged conserved herpesvirus protein kinases (CHPKs)

The CHPKs are grouped as a kinase family based on their conserved genome location and limited sequence homology. While some common functions for select members of the CHPK family have been identified, a thorough functional comparison of this group of kinases has not been reported. Upon the revelation that the HHV-5 (HCMV) CHPK, the UL97 protein, was a viral Cdk ortholog [46], we initiated experiments to determine if the other CHPKs were also v-Cdks. We obtained an expression plasmid for the HHV-2 CHPK which, upon transfection into mammalian cells, produces an active kinase that is tagged with the hemagglutinin (HA) epitope [48]. We then generated individual plasmids that express HA epitope-tagged derivatives of the other seven CHPKs (Table 2). Previous reports indicate that epitope-tagging does not eliminate the kinase activity of the seven CHPKs for which such an analysis has been conducted [48], [49], [50], [51], [52], [53], [54], [55]. The activity of an epitope-tagged CHPK of HHV-7 has not been previously examined. Verification of the correct CHPK sequence by direct analysis (data not shown) and our finding that each tagged kinase scored positive in at least one activity assay (Table 3) provide confidence that our expression plasmids produced active kinases. Although we cannot rule out changes in substrate specificity or specific activity due to the epitope tag, we do note that tagged CHPKs can complement the growth defects of HHV-4 and HHV-5 mutant viruses lacking the untagged (wild type) CHPK gene [52], [56]. Importantly, the epitope tag allows us to monitor the expression and localization of these eight different kinases with the same antibody. Individual transfection of these eight plasmids into human U-2 OS cells allowed for the production of the viral proteins which, when detected on Western blots with an HA antibody, migrated near the predicted molecular weight (Table 2) of the full-length protein [34], [35], [57], [58], [59], [60], [61]. Adjusting the amount of transfected plasmid DNA allowed us to identify experimental conditions under which the steady state protein levels achieved for six of these eight different proteins was consistent and comparable. However, the CHPKs encoded by HHV-2 and HHV-5 often accumulated to lower levels. Therefore, Western blot expression controls are presented for each individual experiment.

**Table 3:** Summary of CHPK activities analyzed in this study.

CHPK	Rb				Lamina		CDK	PML-NB	Aggresomes	
	Sh	807	780	821	Disruption	P-Ser-22			Nuclear	Cyto
**1**	−	−	−	−	17±5P = 0.14	5.9±0.09P = 0.08	29±2P = 0.49	9.8±1.4P = 0.46	**83±2** **P = 0.016**	79±10P = 0.19
**2**	−	−	−	−	**31±5** **P = 0.0098**	42±17P = 0.06	33±6P = 0.10	7.2±1.3P = 0.11	**41±3** **P = 0.0013**	**44±7** **P = 7.9E-5**
**3**	−	−	−	−	12±1P = 0.30	6.7±1.6P = 0.44	34±6P = 0.22	9.2±1.0P = 0.35	**81±2** **P = 0.0098**	84±8P = 0.43
**4**	**+**	**+**	**+**	**+**	**70±7** **P = 0.0008**	**82±6.3** **P = 0.002**	**50±4** **P = 0.002**	**4.4±1.6** **P = 0.04**	40±25P = 0.065	**32±15** **P = 0.0025**
**5**	**+**	**+**	**+**	**+**	**72±4** **P = 3.4E-5**	**71±5.6** **P = 0.002**	**42±9** **P = 0.047**	7.0±1.2P = 0.10	**70±5** **P = 0.015**	**56±22** **P = 0.046**
**6**	**+**	**+**	**+**	**+**	**68±7** **P = 0.0010**	**80±1.4** **P = 3.2E-7**	**49±5** **P = 0.006**	8.5±2.0P = 0.26	**86±2** **P = 0.03**	86±5P = 0.66
**7**	**+**	**+**	**+**	**±**	**58±0.6** **P = 0.0016**	**75±3.3** **P = 5.2E-5**	**37±4** **P = 0.009**	8.3±1.9P = 0.23	91±4P = 0.39	88±5P = 0.98
**8**	**+**	**+**	−	−	**86±3** **P = 6.5E-6**	**71±3.2** **P = 5.9E-5**	38±10P = 0.10	6.6±2.2P = 0.09	93±4P = 0.97	86±6P = 0.64
**EV**	−	−	−	−	9.9±3	3.2±1	29±3	11±3.1	93±0.7	88±6

Results presented are displayed with standard deviation and P values (where applicable). A positive result for an individual activity is shown with bolded text. Rb phosphorylation activities were determined qualitatively as readily apparent (+), marginal (±), or absent (−). For every other assay, assignment of functional activity required statistical significance (P<0.05), and the experimental values obtained for the CHPK-positive cells are shown. Abbreviations are as follows: EV, no kinase control; 1–8, CHPK of the indicated human herpesvirus; Sh, Rb phosphorylation detected by electrophoretic mobility shift; 807, 780, 821, Rb phosphorylation demonstrated with antibodies that detect Rb only when phosphorylated on the indicated amino acid residue; Disruption, percentage of cells with disrupted nuclear lamina; P-Ser-22, percentage of cells staining for lamin A phosphorylated on serine 22; CDK, percentage of budded cells in the yeast complementation assay; PML-NB, average number of PML-NBs; Nuclear, percentage of cells containing nuclear aggresomes; Cyto, percentage of cells containing cytoplasmic aggresomes.

**Figure 1:**
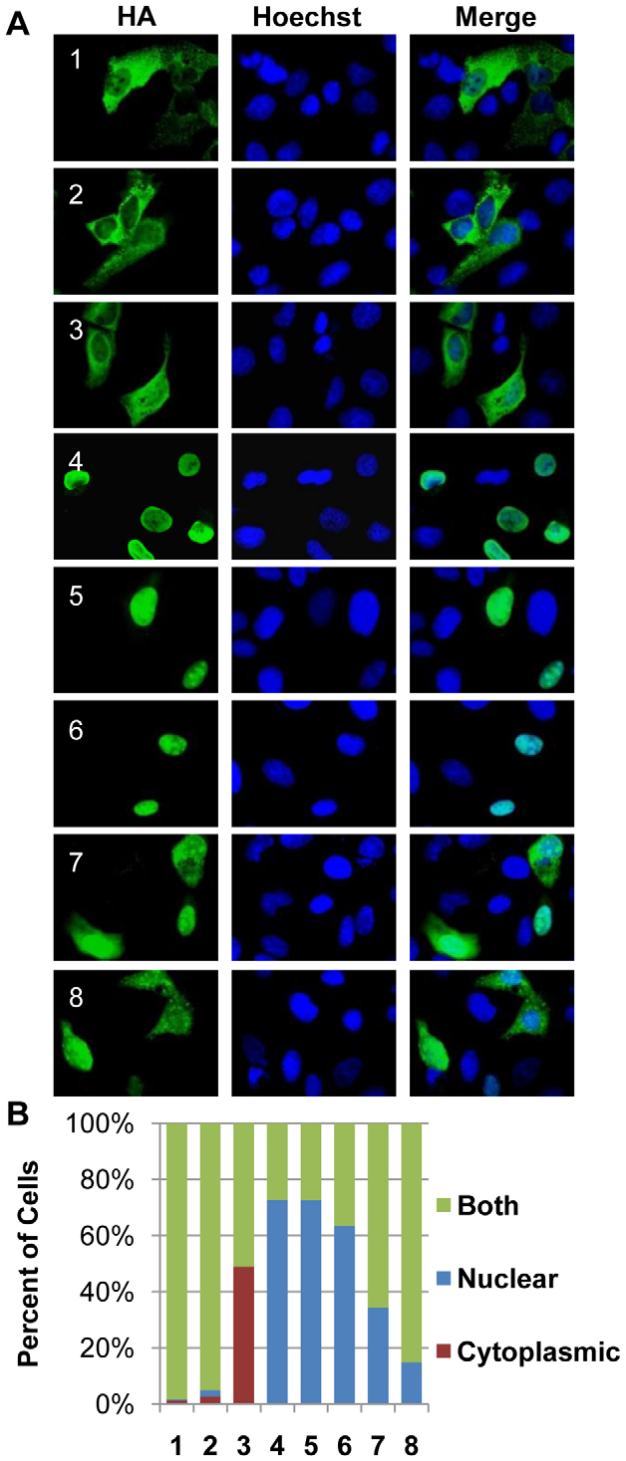
Localization of the CHPKs in U-2 OS cells. (**A**) U-2 OS cells plated on coverslips were transfected with expression plasmids encoding each of the eight CHPKs (denoted by their human herpesvirus number 1-8). Intracellular localization at 24 hours post transfection (hpt) was analyzed by indirect immunofluorescence microscopy after staining with anti-HA antibody (green) and counterstaining nuclei with Hoechst (blue). (**B**) At least 300 kinase positive cells from the experiment described above were scored for CHPK localization. The percentage of cells displaying the indicated localization pattern (primarily nuclear, primarily cytoplasmic, or both nuclear and cytoplasmic) for each CHPK (1–8) is shown.

The sub-cellular localization of the transfected CHPKs in U-2 OS cells was examined by fluorescence microscopy (Fig. 1A) and quantitated (Fig. 1B) by visually determining the percentage of cells with primarily nuclear localization of the kinase, primarily cytoplasmic localization, or localization to both cellular compartments. All of the kinases were found in both the nucleus and cytoplasm in at least 25% of the transfected cells. The alpha-herpesvirus CHPKs (HHV-1, -2, and -3) generally showed both strong cytoplasmic and faint nuclear staining. Certain members of the beta- and gamma-herpesvirus CHPKs showed predominantly nuclear staining (HHV-4, -5, and -6), while others (HHV-7 and -8) were more likely to be found in both cellular compartments. These observed sub-cellular localizations are in agreement with previously published data [34], [35], [59], [60], [62], [63].

### Expression of the beta- and gamma- CHPKs leads to Rb phosphorylation

The retinoblastoma (Rb) and lamin A/C proteins are phosphorylated by the cyclin-dependent kinases (Cdks) that control cell cycle progression. Rb is a tumor suppressor responsible for regulating the G1/S cell cycle checkpoint [64]. Phosphorylation of Rb on specific Cdk-consensus sites results in the dissociation of protein complexes between Rb, histone deacetylases (HDACs), and E2F transcription factors and allows for the expression of E2F-responsive genes that drive progression through G1 and entry into the S-phase of the cell cycle [64]. Certain E2F-responsive genes also play critical roles in nucleotide biosynthesis and DNA replication. Many DNA viruses partially rely on cellular machinery for the replication of their genomes, and therefore target Rb for inactivation during infection [65], [66]. For example, during HHV-5 (HCMV) infection, the UL97 CHPK is necessary and sufficient for Rb inactivation through phosphorylation on Cdk consensus sites [46]. Rb inactivation appears to be a critical function of UL97 for efficient viral replication [67].

**Figure 2:**
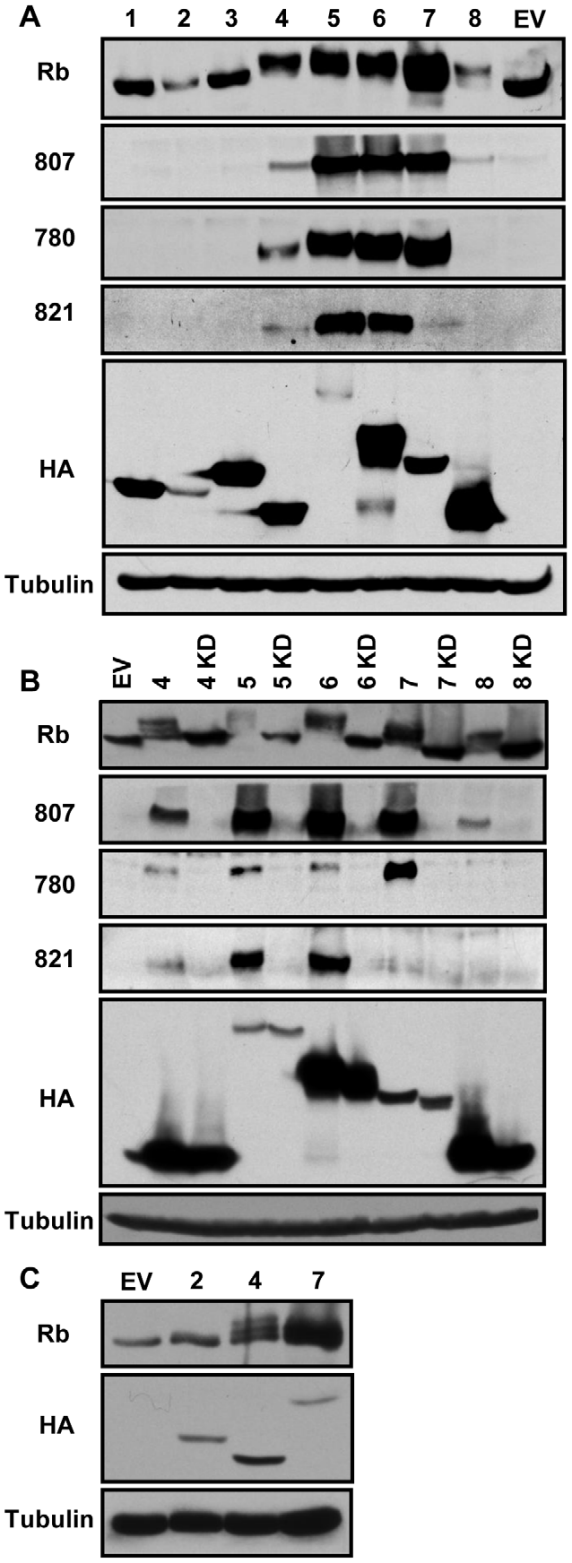
The beta- and gamma-herpesvirus CHPKs phosphorylate Rb. (**A**) Lysates from Saos-2 cells transfected with plasmids encoding Rb and either an empty vector (EV) or one encoding the indicated human herpesvirus (1–8) CHPKs were harvested 48 hpt and analyzed by Western blot with the indicated antibodies. Rb, total Rb; 807, Rb phosphorylated on serine residues 807 and 811; 780, Rb phosphorylated on serine 780; 821, Rb phosphorylated on threonine 821; HA, epitope tag; Tubulin, tubulin loading control. (**B**) Both wild type and kinase-deficient (KD) alleles of the beta- and gamma-herpesvirus CHPKs (4–8) were analyzed in the Saos-2 Rb phosphorylation assay as described above. (**C**) Lower levels of expression plasmids for the HHV-4 and HHV-7 CHPKs were analyzed in the Saos-2 Rb phosphorylation assay as described above. Please see the Materials and Methods section for details.

*In vivo* phosphorylation of Rb by an ectopically-expressed kinase can be easily and reliably determined only in human Saos-2 cells. These Rb-null osteosarcoma cells fail to phosphorylate ectopically-expressed Rb unless a cyclin protein (cellular or viral) or UL97 is included in the transfection [46], [68], [69]. Rb phosphorylation in Saos-2 cells can be observed on Western blots by an electrophoretic mobility shift of the protein to higher molecular weights, as well as with phospho-specific antibodies that detect Rb proteins only when phosphorylated on certain Cdk consensus sites. We found that the CHPKs encoded by all of the beta- and gamma-herpesviruses (HHV-4, -5, -6, -7, and -8) were able to phosphorylate Rb when co-transfected into Saos-2 cells (Fig. 2A). Phosphorylation was detected by both the shift in molecular weight and with three independent phospho-specific antibodies that detect Rb only when phosphorylated on specific Cdk consensus sites that regulate association with the E2F proteins [70], [71]. Interestingly, the beta-herpesvirus CHPKs induced the phosphorylation of Rb-inactivating residues to a substantially higher degree than the gamma-herpesvirus proteins (Fig. 2A). The alpha-herpesvirus proteins (HHV-1, -2, and -3) were unable to phosphorylate Rb (Fig. 2A), as judged by both electrophoretic mobility and the lack of reactivity with the phospho-specific antibodies. The ability of the beta- and gamma-herpesvirus CHPKs to phosphorylate Rb required that each protein was an active kinase. Substitution of the catalytic lysine to create kinase-inactive mutants inhibited the ability of the CHPKs to phosphorylate Rb, shown by both molecular weight shift as well as with the phospho-Rb-specific antibodies (Fig. 2B).

There was no correlation between steady state expression level and the ability to phosphorylate Rb (Fig. 2A). CHPKs that accumulated to high levels either did (e.g. HHV-4 and HHV-7) or did not (e.g. HHV-3) phosphorylate Rb. Likewise, CHPKs that accumulated only to low levels either did (e.g. HHV-5) or did not (e.g. HHV-2) phosphorylate Rb. To further explore any potential relationship between expression level and Rb phosphorylation, we decreased the amount of HHV-4 and HHV-7 CHPK expression plasmid transfected in an attempt to equalize, as much as possible, the steady-state expression levels of these Rb-phosphorylating kinases to the non-Rb-phosphorylating HHV-2 kinase. We found that lower steady state levels of the HHV-4 and HHV-7 CHPK still led to Rb phosphorylation (Fig. 2C), as seen by the shift in apparent molecular weight. While substantial kinase overexpression may result in the phosphorylation of non-physiologic substrates (false-positives), and low expression levels may prevent detection of the phosphorylation of true substrates (false-negatives), the data we present here are consistent with the conclusion that the beta- and gamma-herpesvirus CHPKs, but not the alpha-herpesvirus CHPKs, result in Rb phosphorylation when ectopically expressed.

### The beta- and gamma- CHPKs induce lamin A phosphorylation and lamina disruption

Like Rb, lamin A/C is also a Cdk substrate phosphorylated by the CHPKs of HHV-4 and HHV-5 on Cdk consensus sites [45], [72]. Lamins are intermediate filament proteins that line the inner nuclear membrane as part of the nuclear lamina [73]. During mitosis, the nuclear lamina is disassembled after phosphorylation of lamin A/C by Cdk1 [73]. During viral infections, the nuclear lamina likely represents a physical barrier to herpesviral capsids that must leave the nucleus through envelopment at the inner nuclear membrane. Thus, it has been proposed that in order to gain access to the inner nuclear membrane, herpesviruses must disrupt the nuclear lamina [74]. In cells infected with HHV-1, HHV-4, and HHV-5, lamina disruption is achieved through phosphorylation of lamin A/C on Cdk consensus sites [23], [45], [72].

**Figure 3:**
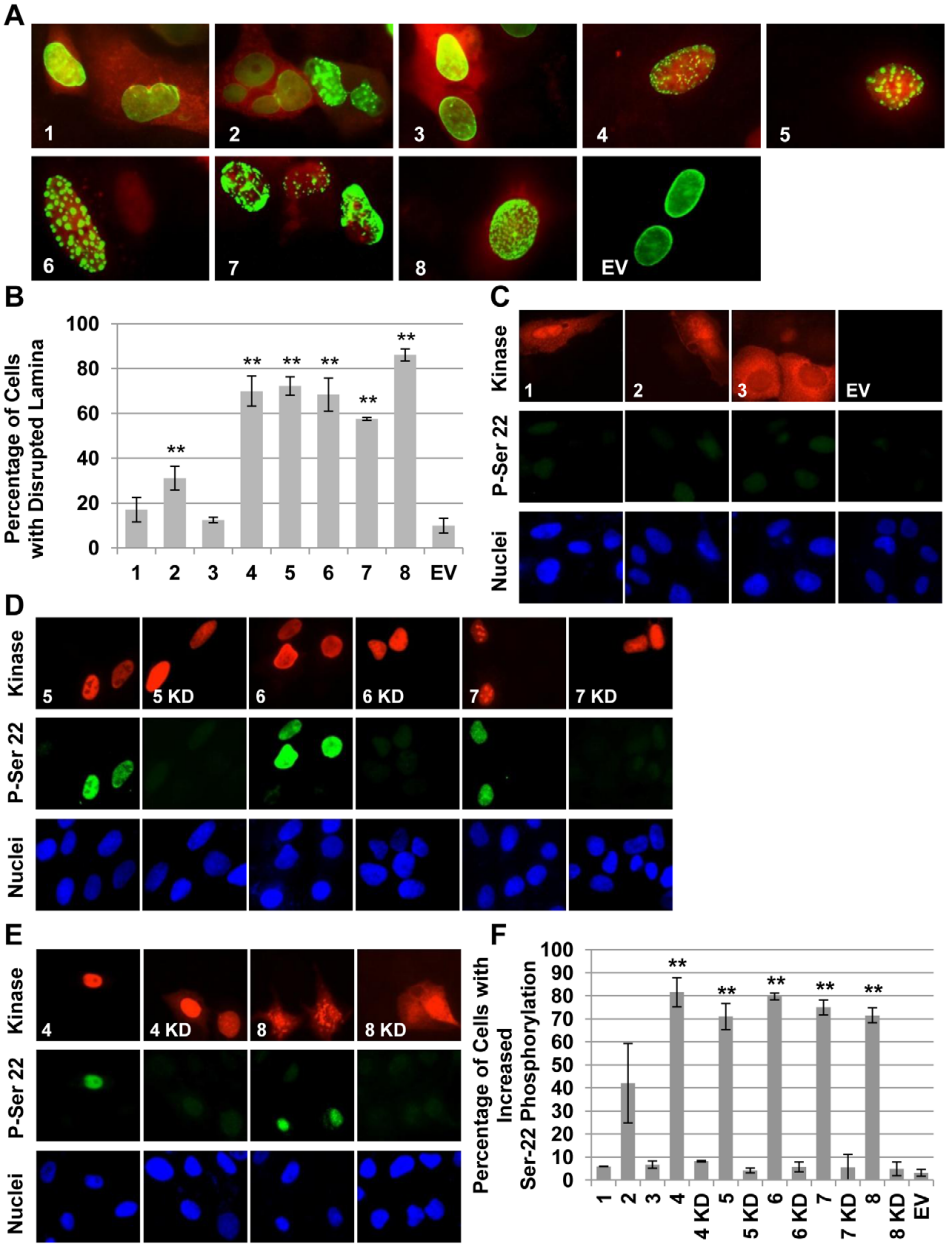
The beta- and gamma- herpesvirus CHPKs disrupt the nuclear lamina and phosphorylate lamin A. (**A**) U-2 OS cells plated on coverslips were co-transfected with the indicated CHPK (1–8) or an empty vector (EV) and a plasmid expressing a GFP-lamin A fusion protein. Coverslips were harvested at 24 hpt, stained for indirect immunofluorescence with an HA antibody and Hoechst (not pictured), and visualized by fluorescence microscopy. Representative images showing GFP-lamin A (green) and the CHPK (red) are displayed. (**B**) At least 300 kinase and GFP-lamin A positive cells from the experiment described above were scored for nuclear lamina disruption. The percentage of cells displaying a disrupted GFP-lamin A signal for each CHPK (1–8) is shown. Error bars represent standard deviation. Double asterisks (**) indicate statistically significant differences from empty vector (EV) transfected cells (P≤0.01). (**C**) U-2 OS cells plated on coverslips were transfected with the indicated alpha-herpesvirus CHPK (1–3) or an empty vector (EV) and then cultured in low serum media. Coverslips were harvested at 36 hpt and stained for indirect immunofluorescence with an HA antibody to detect the CHPK (Kinase), an antibody that detects lamin A only when phosphorylated on Ser-22 (P-Ser 22), and Hoechst (Nuclei). (**D**) Beta-herpesvirus CHPKs (5–7) or their kinase deficient counterparts (KD) were analyzed as described above. (**E**) Gamma-herpesvirus CHPKs (4,8) or their kinase deficient counterparts (KD) were analyzed as described above. (**F**) Cells from the experiments described above (C–E) were scored for lamin A phosphorylation on serine-22. The percentage of cells displaying lamin phosphorylation for wild type (1–8) and KD (4–8) CHPKs is shown. Error bars represent standard deviation. Double asterisks (**) indicate statistically significant differences from empty vector (EV) transfected cells (P≤0.01).

We found that beta- and gamma-herpesvirus CHPKs (HHV-4, -5, -6, -7, and -8), but not those of the alpha-herpesviruses (HHV-1, -2, and -3), were able to efficiently disrupt the nuclear lamina in transfected U-2 OS cells (Fig. 3A). The nuclear lamina was visualized by fluorescence microscopy in cells co-transfected with expression plasmids for the CHPKs and human lamin A fused to GFP. Western blots of the ectopically-expressed proteins are shown in Figure S1. Non-confocal fluorescent images of the transfected cells show a dim GFP signal throughout the body of the nucleus that is brighter where lamin A/C concentrates at the nuclear periphery (see the panel with empty vector co-transfected cells, Fig. 3A, bottom right). Co-expression (visualized by indirect immunofluorescence with an HA antibody) of the beta- and gamma-herpesvirus CHPKs, and to a lesser extent the HHV-2 CHPK, caused a redistribution of GFP from the ring-like signal around the nuclear perimeter into large punctate spots found throughout the nucleus (Fig. 3A) in the majority of cells imaged. For these CHPKs, the percentage of kinase-positive cells showing disrupted nuclear lamina was statistically different than cells transfected with GFP-lamin A and an empty vector control (Fig. 3B). The HHV-1 and HHV-3 alphaherpesvirus CHPKs did not visibly disrupt the nuclear lamina at a frequency distinguishable from empty vector transfected cells.

We also examined the ability of ectopically-expressed CHPKs to direct the phosphorylation of the endogenous lamin A protein at serine-22. Phosphorylation of this and other lamin A residues by cellular Cdk1 initiates the process of lamina breakdown during mitosis [75]. Matching our results from the GFP-lamin experiments (Fig. 3A and 3B), the alpha-herpesvirus CHPKs were unable to induce serine-22 phosphorylation (Fig. 3C and 3F), whereas the beta- (Figure 3D and 3F) and gamma-herpesvirus CHPKs (Fig. 3E and 3F) did induce serine-22 phosphorylation. Kinase-inactive mutants of the beta- and gamma-herpesvirus CHPKs were unable to induce serine-22 phosphorylation. Western blots of the ectopically-expressed CHPKs are shown in Figure S2, and quantitation of the data is shown in Figure 3F.

The HHV-2 CHPK showed low level lamina disruption that was statistically different from an empty vector control (Fig. 3B). A similar level of serine-22 phosphorylation was observed, although the difference from the empty vector control did not reach statistical significance. Thus, although the HHV-2 CHPK may retain some ability to phosphorylate lamin A, (perhaps more efficiently in the tail region) and to disrupt the nuclear lamina [76], we conclude that these activities are, in general, absent from the alpha-herpesvirus CHPKs, but present in the beta- and gamma-herpesvirus CHPKs. Although experiments of this type are often cited as evidence that CHPK-mediated lamin A/C phosphorylation directly leads to lamina disruption, we note that they cannot rule out other mechanisms for lamina disruption upon CHPK expression or herpesvirus infection (such as lamin B or lamin receptor phosphorylation) in addition to or instead of lamin A/C phosphorylation.

### Beta- and gamma-herpesvirus CHPKs function as cyclin dependent kinases (Cdks)

The first human Cdk was cloned by genetic complementation of Cdk mutant yeast cells [77]. Unlike higher eukaryotes, *S. cerevisiae* encodes only a single Cdk, the *CDC28* gene. Yeast cells harboring a temperature-sensitive mutant allele of this gene (*cdc28-13*) exhibit growth arrest in G1 phase upon a shift to the restrictive temperature [78]. Expression of a functional human Cdk in *cdc28-13* cells rescues this arrest phenotype [79]. Expression of the HHV-5 (HCMV) CHPK, the UL97 protein, in *cdc28-13* yeast cells also rescued the cell cycle arrest phenotype at the restrictive temperature, indicating that it has authentic Cdk activity, and identifying UL97 as the first known viral functional ortholog of a Cdk [46].

**Figure 4:**
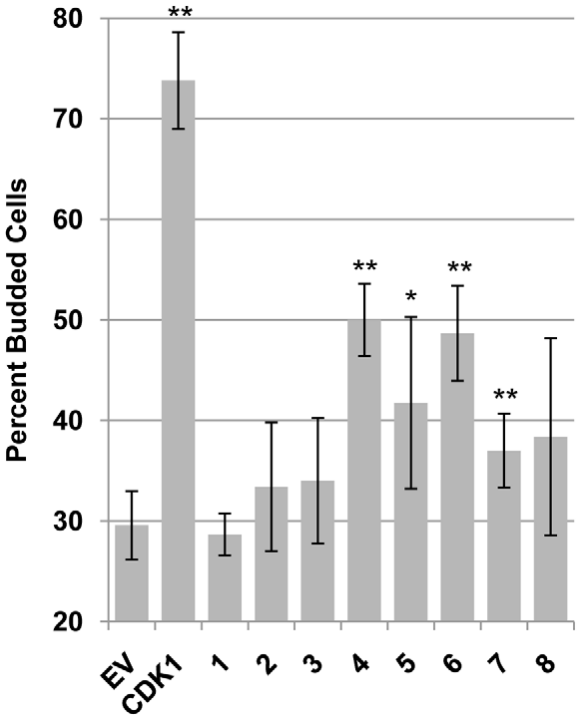
A subset of CHPKs display CDK activity. *S. cerevisiae cdc28-13* strains growing in glucose at the permissive temperature and harboring galactose-inducible expression plasmids for the indicated proteins (Empty Vector, EV; human cyclin-dependent kinase 1, CDK1; CHPKs (1–8)) were transferred to galactose-containing media and shifted to the restrictive temperature for five hours, at which time budded cells (indicative of continued cell cycle progression) were counted by light microscopy. The percentage of budded cells is shown with standard deviation. Asterisks denote statistically significant differences from the empty vector control (* indicates P≤0.05, ** indicates P≤0.01).

We used the same assay to determine if the other CHPKs were also genuine Cdks. Asynchronously growing *cdc28-13* yeast cells harboring plasmids expressing the CHPKs under the control of the GAL1 promoter were shifted to the restrictive temperature to inactivate the sole yeast Cdk, and grown in the presence of galactose to induce the expression of the CHPK. Western blots of the ectopically-expressed CHPKs are shown in Figure S3. Five hours later, cell cycle progression through G1 was verified by determining the number of budded cells. In the absence of an ectopic Cdk, *cdc28-13* cells grown at the restrictive temperature arrested in the G1 phase of the cell cycle as unbudded cells [46], [78], (Fig. 4). However, as was previously observed [46], ectopic expression of human Cdk1, and to a lesser extent the HHV-5 CHPK permitted cell cycle progression through G1 and into S phase of *cdc28-13* cells grown at the restrictive temperature, as evidenced by an increase in the number of budded cells (Fig. 4). This result is indicative of authentic Cdk function. Expression of the other beta-herpesvirus CHPKs (HHV-6 and -7) and the CHPK from the HHV-4 gamma-herpesvirus also rescued the cell cycle defect of *cdc28-13* yeast grown at the restrictive temperature (Fig. 4), indicating that they also have genuine Cdk activity. The HHV-8 gamma-herpesvirus CHPK, the ORF36 protein of KSHV, failed to produce a statistically significant increase in *cdc28-13* budded cells at the restrictive temperature (Fig. 4), although it did display substantial Cdk activity in one of the three experiments performed. Interestingly, KSHV is the only human herpesvirus to encode a viral cyclin [80]. The KSHV cyclin pairs with and activates human Cdks, perhaps obviating the need for Cdk function of the viral CHPK. As expected from their inability to phosphorylate Rb or lamin A/C, the alpha-herpesvirus CHPKs did not display Cdk activity in this assay. Note that we cannot rule out the possibility that the alpha-herpesvirus proteins might not be active when expressed in yeast cells. In total, the Rb, lamin, and yeast experiments functionally define the beta- and gamma-herpesvirus CHPKs as viral cyclin-dependent-like kinases, or v-Cdks.

### The HHV-4 protein (EBV-BGLF4) is the CHPK that most efficiently disrupts PML-NBs

While the CHPKs are grouped as a family based upon their positional homology and limited amino acid similarity, our data indicate that only the beta- and gamma-herpesvirus CHPKs are v-Cdks. These observations indicate that the evolutionary relationship of these eight kinases does not translate to a complete conservation of Cdk-like function. To determine if other functions of these kinases may have been more widely conserved throughout evolution, we screened each kinase for their ability to disrupt PML-NBs, nuclear aggresomes, and cytoplasmic aggresomes, all Cdk-unrelated activities that have been previously attributed to UL97, the HHV-5 (HCMV) CHPK [47].

Promyelocytic Leukemia Nuclear Bodies (PML-NBs) are sub-nuclear structures that appear as multiple punctate spots within the nucleus and are involved in a wide array of cellular activities [81]. During infections with HHV-1, new PML-NBs are generated at the sites where infecting viral genomes enter the nucleus [82]. HHV-5 genomes also co-localize with PML-NBs shortly after infection [83]. Proteins that localize to PML-NBs institute an intrinsic cellular defense against these herpesviruses by silencing viral immediate early (IE) gene expression [84], [85]. Viral proteins delivered to cells upon virion entry and/or expressed as IE genes inactivate this defense and disrupt PML-NBs [86], [87], [88].

**Figure 5:**
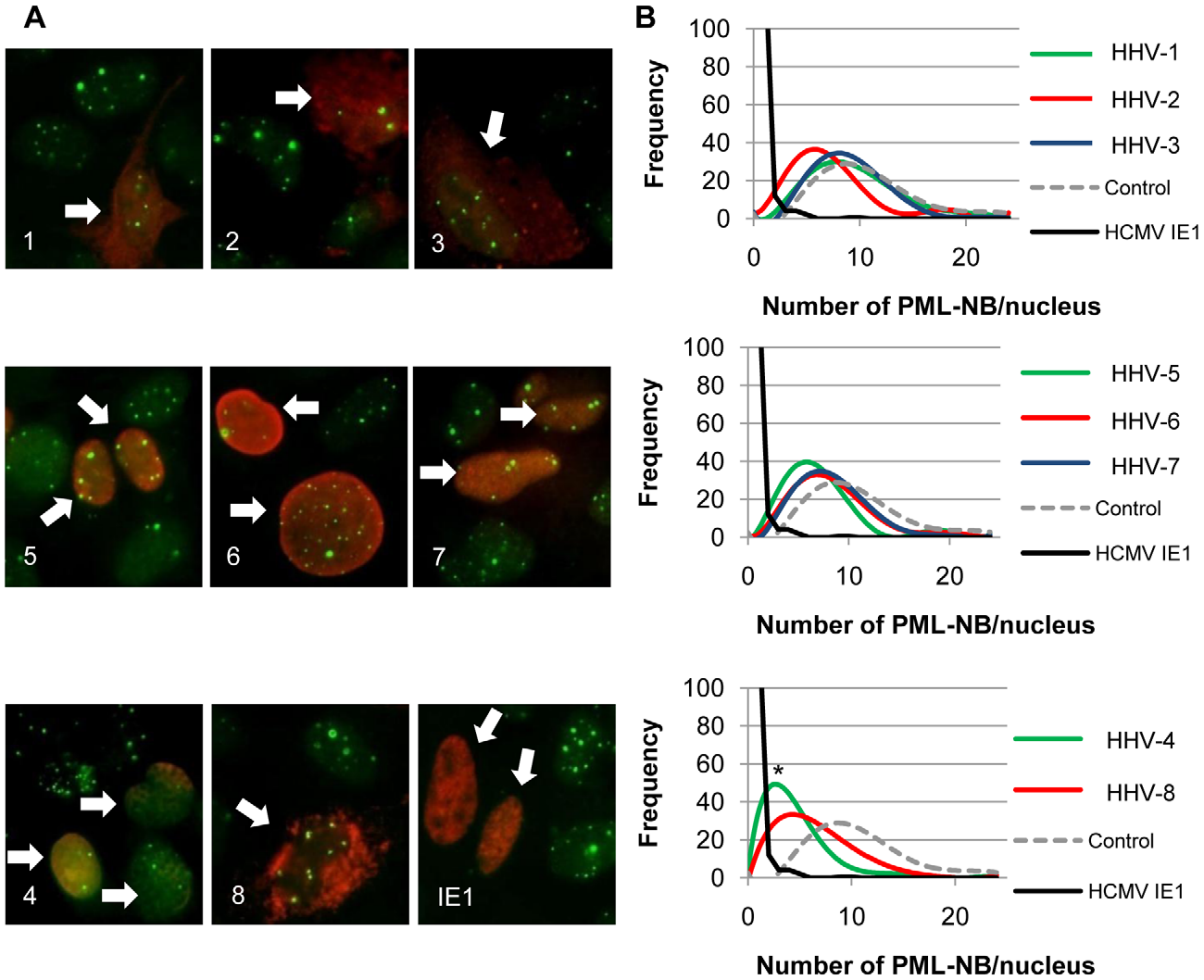
The CHPKs show minor effects on the numbers of PML-NBs per cell. (**A**) U-2 OS cells plated on coverslips were transfected with the indicated CHPK (1–8) or HCMV IE1 and 24 hours later were harvested and stained for indirect immunofluorescence with antibodies for PML, the HA epitope, and Hoechst (not pictured). Representative images showing PML (green) and the CHPK or IE1 (red) are displayed. Arrows denote CHPK/IE1 expressing cells. Images are grouped into the alpha-herpesvirus proteins (top), beta-herpesvirus proteins (middle), and gamma-herpesvirus proteins plus the IE1 control (bottom) (**B**) Using the same coverslips as in A, the number of PML-NB domains was counted in at least 100 kinase positive nuclei for three independent experiments. The frequency with which any specific number of PML-NBs was observed in an individual cell was plotted with Microsoft Excel as a best-fit curve. Control (dashed line) and IE1 expressing cells (black line) are shown in each graph for comparison. The average number of PML-NBs per cell was also determined from this data (see Table 3) and statistically significant differences from control cells are denoted with an asterisk (* indicates P≤0.05). Herpesvirus families are grouped as above.

We used U-2 OS cells and indirect immunofluorescent detection of the PML protein (Fig. 5A) to monitor the effects of ectopically-expressed CHPKs on PML-NBs. Western blots of the ectopically-expressed CHPKs are shown in Figure S4. As expected, we found that the number of PML-NBs in U-2 OS cells resembles a Gaussian distribution between 4 and 24 (Fig. 5B), with an average of 11 per cell (Table 3). Our average agrees well with previously reported data [63]. We used HCMV IE1 as a positive control for PML-NB disruption [86], and counted the number of PML-NBs in 100 CHPK-positive nuclei in three separate experiments. With the exception of HHV-1 and HHV-3, expression of all of the other CHPKs resulted in a perceptible shift in the distribution of PML-NBs per cell towards lower numbers (Fig. 5B). Most of the shifts were small, although the HHV-5 and HHV-8 CHPKs showed more substantial effects. However, only the HHV-4 CHPK caused a drop in the average number of PML-NBs per cell that was statistically different than control cells (Table 3).

### Disruption of nuclear and cytoplasmic protein aggregates by the CHPKs

High synthesis rates, cellular stresses, and other stimuli can lead to protein aggregation and the formation of aggresomes. Ectopic expression of the HHV-5 (HCMV) CHPK (UL97) in uninfected cells has been shown to disrupt aggregates of viral proteins as well as nuclear and cytoplasmic aggresomes formed by GFP-GCP170*, an artificial stimulator of aggresome formation [47], [53]. Conversely, GFP-GCP170* aggregates have been detected in HHV-1 infected cells [89], implying that the HHV-1 CHPK may not disrupt aggresomes.

**Figure 6:**
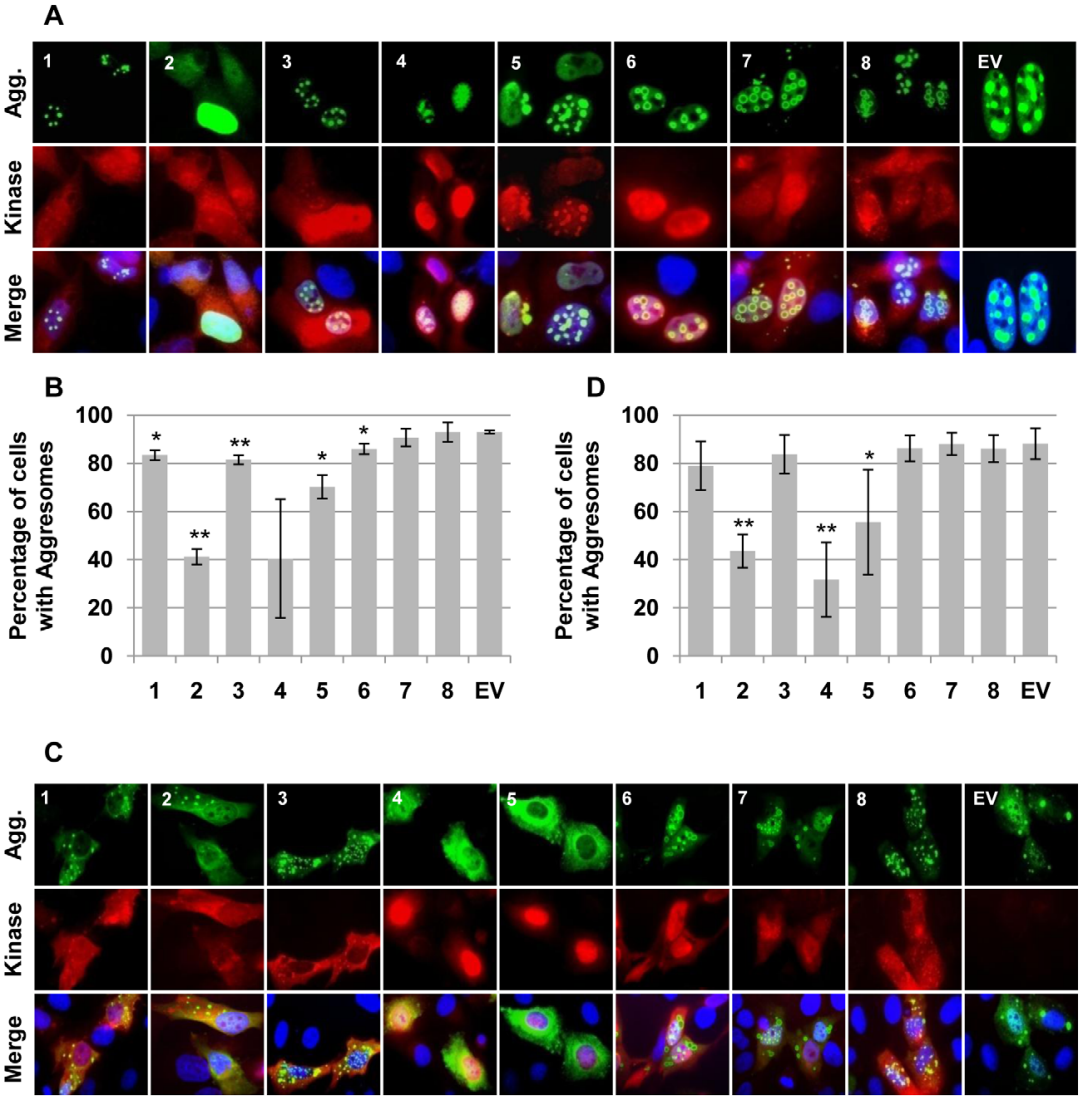
Assorted CHPKs prevent nuclear and/or cytoplasmic aggresome formation. (**A**) U-2 OS cells grown on coverslips were transfected with expression plasmids for Flag epitope tagged Ataxin-Q82 (a spontaneously aggregating protein) as well as the indicated CHPKs (1–8) or an empty vector (EV). Coverslips were harvested 24 hpt and stained for indirect immunofluorescence with antibodies against the Flag epitope, the HA epitope, and Hoechst. Representative images showing Ataxin-Q82 (green), the CHPK (red) and counterstained nuclei (blue) are displayed. (**B**) Using the same coverslips as in A, the number of cells that contained nuclear aggresomes was determined by counting between 200 and 300 Ataxin-Q82 positive cells in three independent experiments. The percentage of kinase and Ataxin-Q82 positive cells that formed nuclear aggresomes is shown with standard deviation. Asterisks denote statistically significant differences from the empty vector control (* indicates P≤0.05, ** indicates P≤0.01). (**C**) Cytoplasmic aggregates formed by the Ataxin-Q82-K772T were imaged as in (A) above. (**D**) Cytoplasmic aggresomes were quantitated as in B above except that four independent experiments were analyzed.

To determine if aggresome disruption was a general feature of the CHPKs, we tested the ability of the kinases to disrupt nuclear or cytoplasmic aggresomes formed by the ataxin-1 protein. Ataxin-1 (Atx1) has a poly-glutamine tract that, when expanded beyond 40 amino acids, produces a protein with a non-native conformation that forms aggregates, is cytotoxic, and causes spinocerebellar ataxia type 1 [90]. We co-transfected cells with the CHPKs and FLAG-tagged derivatives of either Atx1(Q82) or Atx1(Q82)-K772T that form aggregates in the nucleus and cytoplasm, respectively [90]. Western blots of the ectopically-expressed proteins are shown in Figure S5. The presence of at least one aggregate or the total absence of aggregates in ataxin-expressing cells (Fig. 6A and 6C) was determined for at least 200 CHPK-positive cells in at least three independent experiments. We found that the HHV-2 and HHV-5 CHPKs resulted in a statistically significant decrease in the number of cells in which ataxin-1 could form both nuclear (Fig. 6A and 6B) and cytoplasmic (Fig. 6C and 6D) aggregates even though these were the CHPKs expressed to the lowest level (Figure S5). CHPKs encoded by HHV-1, -3, -4, and -6 caused a statistically significant decrease in cells with either nuclear or cytoplasmic aggresomes, but not both (Fig. 6). While the HHV-4 CHPK caused a substantial decrease in the percentage of cells containing nuclear aggresomes, the wide variability in the numbers accumulated over three independent experiments resulted in data that did not achieve statistical significance (Fig. 6B). Our data confirm previous results demonstrating that the HHV-5 CHPK can either prevent or disrupt protein aggregation [47], but also indicate that this ability is not well conserved among the eight CHPKs.

In summary, we found that the most conserved activity of the CHPKs is their Cdk-like function. However, this is only found in the beta-and gamma-herpesvirus members, and is absent in the alpha-herpesvirus proteins. The non-Cdk-like activities reported for the HHV-5 CHPK, the HCMV UL97 protein, are poorly conserved among the other family members. Thus the functional conservation between these eight kinases as a whole, much like their amino acid sequence similarity, is limited.

## Discussion

Cellular substrates of selected CHPKs that are normally phosphorylated by the Cdks in uninfected cells have been identified. These include Rb [46], [47], lamin A/C [45], [72], translation elongation factor 1 delta [49], the carboxy-terminal domain of RNA Polymerase II [91], the helicase complex component MCM4 [92], the condensin protein involved in chromatin packaging [93], and the Cdk inhibitor p27 [51]. However, only the CHPK encoded by HHV-5, the HCMV UL97 protein, had been shown to be a functional Cdk ortholog [46]. In this study, we examined the remainder of the CHPKs for Cdk-like activity, as well as non-Cdk-like activities previously reported for UL97. We employed the yeast complementation assay because it is the most basic and stringent test for Cdk activity, and the Saos-2 assay because it is the most widely-accepted assay for demonstrating Rb phosphorylation *in vivo* by a co-transfected protein. For all other experiments, we used U-2 OS cells because they transfect well, do not express a transforming viral oncogene, and represent a generic human cell (the differing cellular tropisms of the eight human herpesviruses prevented us from using a more physiologically relevant cell type). Effects on cellular targets or processes (as opposed to viral ones) were analyzed so that activity against identical (as opposed to just homologous) substrates could be monitored.

Our results indicate that Cdk-like activity is absent in the alpha-herpesvirus CHPKs, as they were unable to induce Rb or lamin A phosphorylation, lamina disassembly, or yeast *cdc28-13* cell cycle progression. These results agree well with the observations that Rb is not phosphorylated in cells infected with HHV-1, HHV-2, or HHV-3 [66], but is phosphorylated in cells infected with HHV-4 and HHV-5, [94], [95], representative members of the beta- and gamma-herpesvirus families. Our lamin results are also mostly consistent with previously published data. Although it is clear that lamin A/C becomes phosphorylated in cells infected with representative members of the alpha- [23], beta- [45], and gamma- [72] herpesviruses, different kinase families appear to be responsible for these phosphorylation events. A recent analysis [72] found that, in addition to the HHV-4, HHV-5 [96], and HHV-8 CHPKs, the HHV-1 CHPK was able to disrupt the nuclear lamina in HeLa cells [72], however, we detected no activity of the HHV-1 protein in two independent lamin assays (Fig. 3). It is unclear if expression of the papillomavirus E7 oncoprotein in HeLa cells, a known stimulator of Cdk activity [97] that is not present in the U-2 OS cells used here, could explain this difference between the two studies. Thus, although previous studies [72], [76] and our own data with the HHV-2 CHPK (Fig. 3) indicate that the alpha-herpesvirus CHPKs may retain some ability to phosphorylate lamin A/C and to disrupt the nuclear lamina, we suspect, as others have shown, that the members of the alpha-herpesvirus-specific Us3 family of kinases are largely responsible for this activity in virus-infected cells [21], [22], [23], [98]. Thus, we conclude that the alpha-herpesvirus CHPKs do not mimic cellular Cdk activity. Interestingly, cellular Cdks are active during, and required for efficient alpha-herpesvirus replication [66]. The critical substrates for cellular Cdks in alpha-herpesvirus infected cells remain to be determined.

In contrast, the beta- and gamma-herpesvirus CHPKs were found to possess Cdk-like activity. All of these proteins phosphorylated Rb and disrupted the nuclear lamina, and all but one rescued cell cycle progression in the yeast complementation assay. It is presently undetermined what (if any) advantage encoding and expressing their own Cdk-like protein affords these viruses that utilization of cellular Cdk activity would not. Nevertheless, this analysis, along with our previous study [46] identifies the HHV-4 (EBV-BGLF4), HHV-5 (HCMV-UL97), HHV-6 (U69), HHV-7 (U69), and HHV-8 (KSHV-ORF36) CHPKs as virally-encoded cyclin-dependent-like kinases, or v-Cdks.

Unlike cellular Cdks, the HHV-5 CHPK (HCMV UL97), the first identified v-Cdk, lacks many of the regulatory features that restrict kinase activity during certain stages of the cell cycle or under physiological stresses [46]. For example, UL97 lacks conserved sequences for cyclin binding and appears to have cyclin-independent kinase activity. UL97 has an amino acid substitution at the Cdk site (Thr 160 of Cdk2) of phosphorylation by Cdk activating kinase (CAK), and is active under conditions of CAK inhibition. UL97 lacks amino acid residues important for binding both the Cip/Kip and INK classes of cyclin-dependent kinase inhibitors, and is immune to inhibition by p21^WAF1/CIP1^ *in vivo* and *in vitro*. Finally, UL97 has a phenylalanine substitution for a tyrosine residue (Tyr 15 of Cdk2) found in Cdks which, when phosphorylated in G2 phase or in response to DNA damage, attenuates kinase activity.

**Figure 7:**
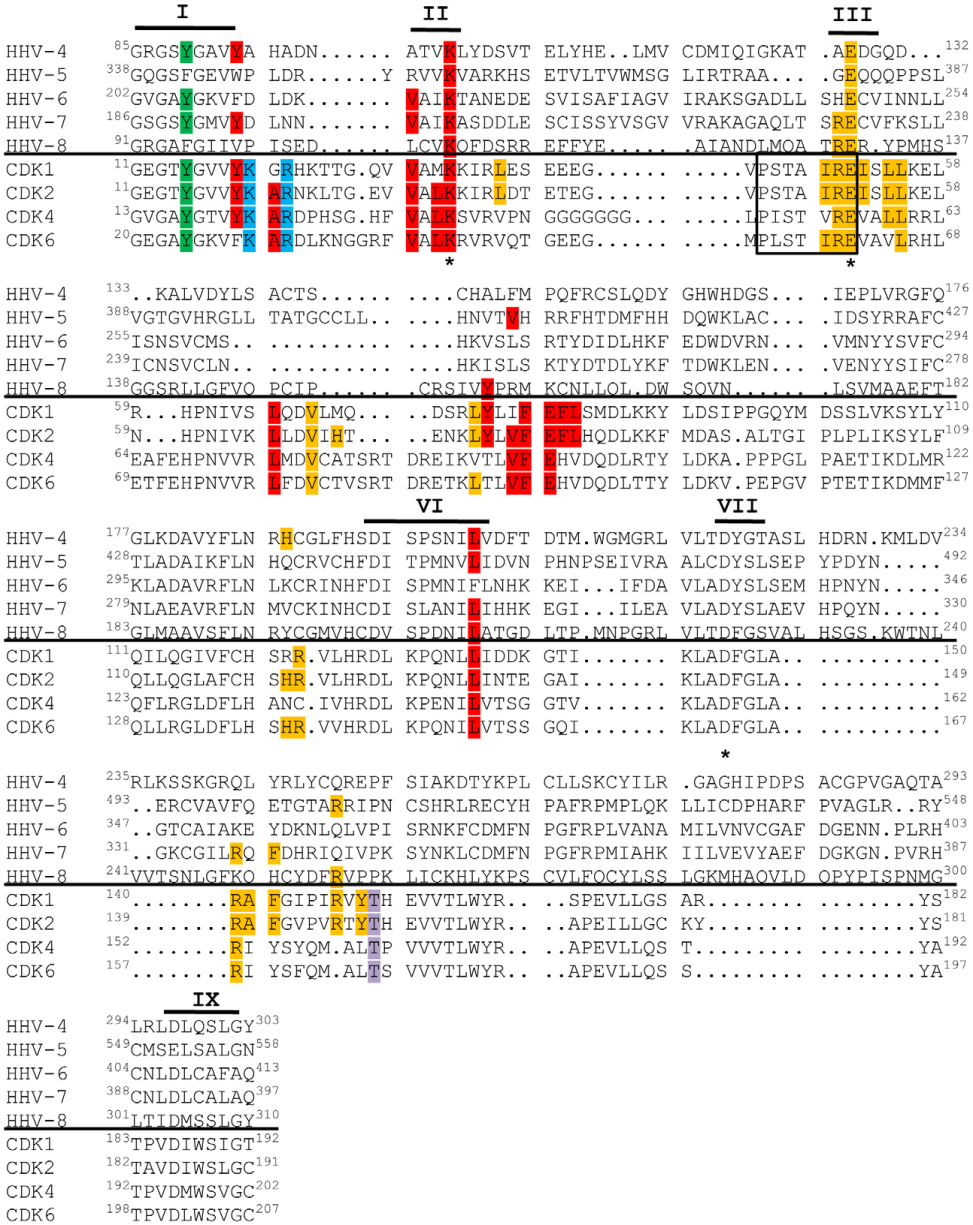
v-Cdks lack conserved cellular Cdk residues that allow for the regulation of kinase activity. The amino acid sequences of the kinase domains of the indicated viral (top) and human (bottom) Cdks were aligned. Conserved kinase subdomains are indicated with Roman numerals [118]. The residue corresponding to tyrosine 15 of Cdk2 is shown in green. The residue corresponding to threonine 160 of Cdk2 is shown in lavender. Amino acids that mediate cellular Cdk binding to p27 [119], a member of the Cip/Kip class of cyclin-dependent kinase inhibitors, are shown in red. Amino acids that mediate cellular Cdk binding to p16 [120], a member of the INK family of cyclin-dependent kinase inhibitors, are shown in blue. The cyclin-binding PSTAIRE sequence in the cellular Cdks is boxed [121], and other residues shown to be important for CDK2 binding to Cyclin A are shown in orange [121]. The catalytic triad residues are marked with asterisks (*) [118].

A sequence alignment of the other v-Cdks showed that, like UL97, none of these kinases contain the cyclin-binding, cyclin-dependent kinase inhibitor-binding, or CAK phosphorylation motifs found in the cellular Cdks (Fig. 7). Thus, the newly-identified v-Cdks lack many of the amino acids and protein domains responsible for regulating the activity of their cellular functional orthologs. Interestingly, all of the v-Cdks except UL97 and KSHV ORF36 (the HHV-8 CHPK) do contain the tyrosine residue that, in cellular Cdks, allows for the attenuation of kinase activity upon phosphorylation. Whether or not this amino acid substitution affects kinase activity during viral infection remains to be examined.

In the past, designation as a Cdk required not only sequence and functional homology to known Cdks, but also experimental evidence that the activity of the kinase was dependent upon association with a cyclin regulatory subunit [99]. However, a recent nomenclature adjustment has grouped all human and murine kinases with sequence and functional similarity to Cdks into the Cdk family, even if cyclin binding has not been observed or is not expected [100]. Thus, while the beta- and gamma-herpesvirus CHPKs do not appear to require cyclin binding for activity, describing these proteins as v-Cdks is in accordance with the accepted classification system because they are related to true Cdks by amino acid sequence (Fig. 7), and because they mimic Cdk activity (Fig. 2, Fig. 3, Fig. 4, Table 3) even though they are likely not regulated in an identical fashion to cellular Cdks.

The lack of conservation of Cdk activity in the alpha-herpesvirus CHPKs was surprising based on the strong evolutionary relationship of this family of proteins, and prompted us to ask if non-Cdk-like functions were more conserved throughout the CHPK family. Interestingly, we found that non-Cdk-like functions were even less well conserved than Cdk activity (Table 3). Only the HHV-4 CHPK mediated a decrease in PML-NB numbers per cell that reached statistical significance, although other kinases, most notably the HHV-5 and HHV-8 CHPKs, clearly appeared to affect these structures. This finding varies considerably from previous reports, perhaps because of different experimental approaches. Our study used a version of the HHV-4 CHPK tagged at the amino-terminus with the 9 amino acid HA epitope tag, while a previous examination of this protein that failed to demonstrate PML-NB disruption [63] used a 69 amino acid SPA tag at the carboxy-terminus. The differences in tag size and location may affect kinase activity, which was not directly examined in the other study [63], but has been demonstrated here (Fig. 2). A study that concluded the HHV-5 CHPK disrupted PML-NBs [47] was conducted in Cos7 cells that express the simian virus 40 (SV40) tumor (T) antigen, and monitored Sp100 localization as a measure of PML-NBs. Our study visualized PML, the sole protein required for PML-NB formation, in U-2 OS cells that do not express a transforming viral oncogene. It is currently unknown which if any of these differences may explain why the subtle effects on PML-NB distribution that we observed upon expression of the HHV-5 CHPK failed to reach statistical significance.

Proteins that efficiently disrupt PML-NBs (HCMV IE1 and EBV Zta) are expressed within infected cells *in vitro* prior to the time when the CHPKs are expressed [101], [102], so the ability of the HHV-4 and HHV-5 CHPKs to decrease the numbers of PML-NBs when ectopically expressed may have limited relevance during viral infection. Furthermore, because the proteins that localize to PML-NBs and not the PML-NB structures themselves appear to be the mediators of an intrinsic antiviral defense [84], [85], the significance of the ability of these viral proteins to reduce the number of, but not eliminate PML-NBs from cells is unclear, especially since it is unknown whether or not any PML-NB resident proteins are direct substrates of these kinases. Therefore, perhaps unsurprisingly, the ability to disrupt PML-NBs appears to be an activity not well conserved throughout the CHPK family.

Disruption of protein aggregates termed aggresomes is another non-Cdk function attributed to the HHV-5 CHPK. Aggresomes have been hypothesized to represent a cellular anti-viral defense that identifies, initially sequesters, and ultimately disposes of viral proteins to inhibit viral replication [103]. Thus, aggresome disruption could potentially enhance viral infections. However, aggresome formation is also thought to sequester misfolded proteins and perhaps facilitate their degradation by the proteasome or through autophagy [103]. In addition, viruses may commandeer aggresome formation pathways to help form replication or assembly compartments [103]. In these respects, aggresome formation would appear to enhance virus replication.

Different human herpesviruses appear to present examples where aggresome formation has opposing effects on viral replication. Structures morphologically resembling aggresomes form in both the nuclei and cytoplasm of cells infected with HHV-1 and -2 (the herpes simplex viruses) or HHV-5 (HCMV). At early times after HHV-1 infection, aggregates containing viral proteins, cellular chaperones, and proteasomes form in the nucleus [104]. These virus-induced chaperone enriched (VICE) domains are proposed to be sites where misfolded proteins are sequestered away from virus replication compartments, perhaps to facilitate either their proper folding or degradation, as well as to prevent the misfolded proteins from triggering cellular processes deleterious to virus infection such as apoptosis or the unfolded protein response [105]. At late times after HHV-2 infection, similar structures form in the cytoplasm [106]. Disruption of these cytoplasmic aggresome-like structures correlates with decreased HHV-2 yields [106], indicating that the integrity of these structures may positively influence viral infection. In contrast, the disruption of aggresomes may facilitate HHV-5 replication. Cells infected with an HHV-5 mutant lacking its CHPK contain nuclear and cytoplasmic aggresome-like structures that are absent during wild type-infection [53], and produce fewer infectious progeny virions than wild-type infection [32]. These findings have led to the hypothesis that aggresomes may represent a cellular anti-viral defense, and that one role for the HHV-5 CHPK during viral infection is to prevent their formation [47].

Only one CHPK from each family (alpha, HHV-2; beta, HHV-5; and gamma, HHV-4) disrupted cytoplasmic aggresomes. However, disruption of nuclear aggresomes was the one activity that could conceivably be considered as conserved among these kinases, even though the magnitude of this disruption was minimal in some cases. All of the three alpha-herpesvirus CHPKs scored positive for this assay, as did two (HHV-5 and HHV-6) of the three beta-herpesvirus CHPKs. While our statistical analysis argues that both gamma-herpesvirus proteins are devoid of this activity, the HHV-4 CHPK clearly appeared to have an effect on these structures.

Because, for the most part, the non-Cdk-like activities we examined do not segregate to specific viral families (as Cdk activity does), we suggest that cellular proteins that are substrates for individual CHPKs, but not the Cdks, may represent a more divergent set of proteins than might be expected based upon the evolutionary relationship of these kinases. Furthermore, our data suggest that the v-Cdks may have an expanded repertoire of substrates as compared to the alpha-herpesvirus CHPKs. This may not be surprising because the alpha-herpesvirus-specific Us3-family kinases appear to assume at least one v-Cdk role, lamina disruption [21], [22], [23], during viral infection. Whether or not a comprehensive analysis of kinase activity against homologous viral proteins would reveal more conservation of function throughout the CHPK family awaits further examination.

An alternative strategy to assay for functional conservation among the CHPKs would be to determine if other members of the kinase family could complement the growth defect observed in viruses lacking their endogenous CHPK. The few experiments that have been performed support the contention that the v-Cdks have a partially overlapping but also extended substrate range when compared to the alpha-herpesvirus CHPKs. Expression of the HHV-5 CHPK from an HHV-1 genome in which its CHPK was deleted resulted in a complete restoration of progeny virion formation at both high and low multiplicities of infection [107], indicating that this v-Cdk can perform all the necessary functions for viral replication normally carried out by the HHV-1 CHPK. However, when expressed from a recombinant adenovirus, the HHV-1 CHPK was unable to rescue the growth of an HHV-5 CHPK null-mutant virus [108]. Interestingly, that same series of experiments demonstrated that the HHV-4 CHPK could partially complement the growth defect of the HHV-5 CHPK mutant virus, indicating that there is substantially more functional overlap within the v-Cdks than between the v-Cdks and the alpha-herpesvirus CHPKs. A comprehensive genetic analysis of inter-virus CHPK complementation should increase our understanding of the evolutionary path that these viral kinases have traveled, as well as helping to define their critical roles during viral infection.

**Figure 8:**
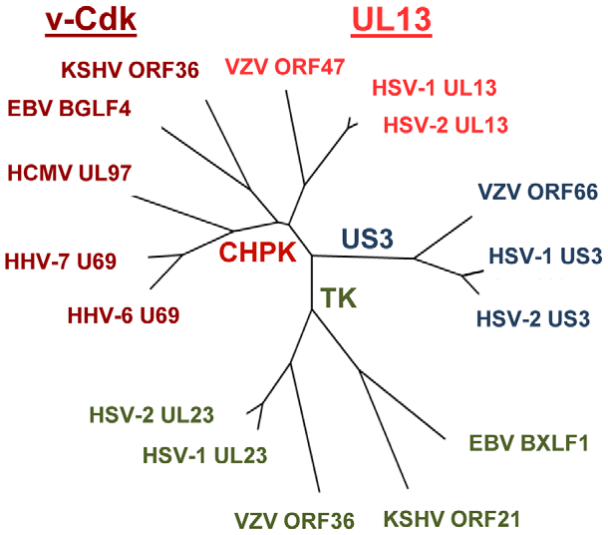
Tree diagram of the human herpesvirus kinases. Amino acid sequence comparison was used to generate a tree diagram showing the interrelatedness of sixteen human herpesvirus kinases. The segregation of the kinases into the Us3, TK, and CHPK families, and the separation of the CHPK family into the UL13 and v-Cdk clades is shown. Line distances represent relative evolutionary separation. The commonly-used names for the human herpesviruses are shown (see Table 2).

In all eight human herpesviruses, the CHPK gene is found in a conserved genomic block and is flanked on either side by homologous genes [109], [110], likely indicating that the current CHPK genes are descended from a single primordial precursor. Over time, the functions of this putative CHPK precursor appear to have diversified differently among these eight viruses. Thus while they represent a single historical kinase family, it may be more practical to consider the CHPK family as representing two individual clades, the UL13s and the v-Cdks. An evolutionary tree diagram of the sixteen shared human herpesvirus kinases (Fig. 8) clearly shows their segregation into different families and clades. Whether the putative primordial CHPK was a Cdk whose Cdk-like activity was lost in the alpha-herpesvirus lineage, or whether it was a kinase that evolved to acquire Cdk-like function in the beta- and gamma-herpesviruses is an interesting topic for speculation and debate.

## Materials and Methods

### Cells and transfections

U-2 OS human osteosarcoma cells were grown in a 5% CO_2_ atmosphere at 37°C in Dulbecco's modified Eagle's medium (Invitrogen) supplemented with 10% (vol/vol) fetal bovine serum (Gemini), 100 U/ml penicillin, 100 ug/ml streptomycin, and 0.292 mg/ml glutamine (Gibco). Cells (2×10^6^) were seeded on 10 cm plates five hours prior to transfection by the calcium phosphate co-precipitation method. After an overnight incubation, the cells were washed twice with medium and then re-fed with serum-containing media (time zero). Different amounts of CHPK expression plasmid DNA were transfected (HHV-2, -5, -7, 15 µg; HHV-1 and -8, 10 µg; HHV-3, 5 µg; HHV-4, 2µg; HHV-6, 1µg) in order to approximately equalize steady state protein levels. Total DNA levels in transfections were balanced with pGEM7 (Promega). Saos-2 cells were grown as above, seeded at a density of 8×10^5^ cells per 60 mm dish, and transfected 5 hours later with TransIT-2020 (Mirus) according to the manufacturer's instructions. Along with 1µg of Rb expression plasmid, different amounts of CHPK expression plasmid DNA were transfected (HHV-2, -5, -5KD, 1.5 µg; HHV-1, -7, -7KD, -8KD, 1 µg; HHV-8, 0.15 µg; HHV-3, -4KD, -6, -6KD, 0.1 µg; HHV-4, 0.05 µg). Transfection conditions for the experiment in Figure 2C were the same except 0.01 µg was transfected for HHV-4, and 0.2 µg for HHV-7.

### CHPK expression plasmids

Kinase alleles (except HHV-2) were amplified by PCR, sequence verified (see Table 2), and subsequently cloned into the pCGN vector that adds an N-terminal hemagglutinin (HA) epitope tag and expresses genes from the HCMV major immediate early promoter. HA-tagged alleles for all of the CHPKs except HHV-5 were also cloned into the yeast expression vector pMSS78 under the control of the yeast *GAL1* promoter [111]. The plasmid that expresses V5 epitope-tagged HHV-5 CHPK in yeast has been previously described [46]. Kinase-deficient (KD) mutants (Table 2) were created by standard mutagenesis approaches and confirmed by complete sequencing of the mutant allele. PCR templates were as follows: HHV-1, viral genomic DNA strain KOS, a gift from Curtis Brandt; HHV-3, plasmid pCAGGS ORF47.12 [50], a gift from Charles Grose; HHV-4, plasmid pcDNA3.1-FLAG BGLF4 [112], a gift from Thomas Stamminger; HHV-5, ppUL97-V5 [53], a gift from Mark Prichard; HHV-6 and HHV-7, cosmids pMF147-19 [113] and I6 (unpublished) respectively, gifts from Steven Dewhurst; HHV-8, plasmid pND ORF36 [55], a gift from Paul Luciw. Plasmid pSG5-UL13 3′ HA [48] kindly provided by Lynda Morrison, was used to express a kinase-active C-terminally tagged HHV-2 CHPK from the SV40 promoter.

### Sequence alignment and phylogenic tree assembly

The sequence comparison was created with a ClustalW alignment in MEGA 4 [114], and utilized only the kinase domains of the indicated proteins. The parameters were as follows: Pairwise alignment - gap opening penalty 2, gap extension penalty 0.05; multiple alignment - gap opening penalty 5, gap extension penalty 1, utilizing the Blosum matrix. The alignment was heuristically adjusted to align kinase domain III. The phylogenic tree was constructed by the neighbor-joining method, in MEGA 4 [114].

### Western blots, indirect immunofluorescence, and antibodies

Equal amounts of protein (determined by Bradford assay) from cell lysates prepared in radioimmunoprecipitation assay (RIPA) buffer with protease inhibitors were analyzed by Western blotting (WB) as previously described [115]. Cells grown on glass coverslips were processed for indirect immunofluorescence (IF) as previously described [116]. Images were produced with a Nikon Eclipse TE2000-S or Zeiss Axiovert 200M microscope. The following antibodies were from commercial sources: HA (IF: Roche 3F10; WB: Covance MMS-101P), PML (Santa Cruz sc-966), Total Rb (Cell Signaling 9309), Rb p-Ser807/811 (Cell Signaling 9308), Rb p-Ser780 (Cell Signaling 9307), Rb p-Thr821 (Biosource 44-582G), Lamin A/C (Novocastra NCL-Lam-A/C) Lamin A p-Ser22 (Cell Signaling 2026), Flag (Sigma F1804), V5 (Invitrogen R960-25) Tubulin (DM 1A Sigma), PGK (Invitrogen 459250). The HCMV IE1 antibody 1B12 has been previously described [117].

### Assays

Rb Phosphorylation in Saos-2 cells and Cdk activity in *S. cerevisiae cdc28-13* cells were determined as previously described [46]. For the nuclear lamina disruption assays, U-2 OS cells grown on coverslips were co-transfected with expression plasmids for the indicated CHPK and pEGFPhLA-WT [72] that expresses a GFP-lamin A fusion protein (a kind gift of David Gilbert). Coverslips were harvested 24h later, stained for the CHPK, and then visualized by fluorescence microcopy. At least 300 CHPK- and GFP-lamin A-positive cells for each co-transfection were analyzed in three independent experiments. For the lamin A/C serine-22 phosphorylation assay, U-2 OS cells grown on coverslips were transfected with expression plasmids for the indicated CHPK. Cells were then incubated in media containing 0.1% FBS for 36 hours before harvesting the coverslips, staining for the CHPK and lamin A phosphorylated at serine-22, and visualization by fluorescence microscopy. At least 200 (wild type) or 100 (kinase-deficient) CHPK-positive cells were analyzed in three separate experiments. For PML-NB disruption assays, U-2 OS cells grown on coverslips were transfected with an expression plasmid for the indicated CHPK or pCGN-IE1 [117]. Coverslips were harvested 24h later, stained for PML and the CHPK, and then visualized by fluorescence microscopy. The number of PML-NBs in 100 individual CHPK- or IE1-positive cells was counted in each of three independent experiments. For the aggresome disruption assays, U-2 OS cells grown on coverslips were co-transfected with expression plasmids for the indicated CHPK and either p3PK-Flag-Ataxin-1 or p3PK-Flag-Ataxin-1 K722T [90] that express, respectively, nuclear or cytoplasmic versions of the ataxin-1 Q82 protein that spontaneously forms large aggregates fused to GFP (kindly provided by Harry Orr). Coverslips were harvested 24h later, stained for the CHPK, and visualized by fluorescence microscopy. At least 200 (nuclear) or 300 (cytoplasmic) CHPK- and ataxin-positive nuclei were analyzed in three (nuclear) or four (cytoplasmic) separate experiments.

## Supporting Information

Figure S1 Western blot analysis of lysates from the lamina disruption experiment presented in Figure 3A. Lysates from the transfected U-2 OS cells shown in Figure 3A were analyzed by Western blot with the indicated antibodies. Note that these cells express both endogenous lamin A as well as the ectopic lamin A-GFP fusion protein, and both are recognized by the lamin A antibody. The HA antibody recognizes the CHPKs, and tubulin serves as a loading control. Numbers represent the different human herpesvirus CHPKs. EV, empty vector.
(0.23 MB TIF)

Figure S2 Western blot analysis of lysates from the lamin A phosphorylation experiment presented in Figures 3C, 3D, and 3E. Lysates from the transfected U-2 OS cells shown in Figure 3C, 3D, and 3E were analyzed by Western blot with the indicated antibodies. The HA antibody recognizes both wild type and kinase deficient (KD) CHPKs, and tubulin serves as a loading control. Numbers represent the different human herpesvirus CHPKs. EV, empty vector.
(0.36 MB TIF)

Figure S3 Western blot analysis of lysates from the *S. cerevisiae* Cdk complementation asay shown in Figure 4. Lysates from *S. cerevisiae* harboring plasmids expressing the indicated kinase and treated with galactose were analyzed by Western blot with the indicated antibodies. The HA antibody recognizes all CHPKs except for the HHV-5 protein, which is visualized with the V5 antibody. Cdk1 expression was not analyzed. PGK (3-Phosphoglycerokinase) serves as a loading control. Numbers represent the different human herpesvirus CHPKs. EV, empty vector.
(0.22 MB TIF)

Figure S4 Western blot analysis of lysates from the PML-NB disruption experiment presented in Figure 5. Lysates from transfected U-2 OS cells shown in Figure 5A were analyzed by Western blot with the indicated antibodies. The HA antibody recognizes the CHPK, and tubulin serves as a loading control. The approximate localization of molecular weight markers (in Kilo Daltons, kDa) is displayed. Numbers represent the different human herpesvirus CHPKs.
(0.20 MB TIF)

Figure S5 Western blot analysis of lysates from the aggresome disruption experiments presented in Figure 6. (A) Lysates from transfected U-2 OS cells shown in Figure 6A were analyzed by Western blot with the indicated antibodies. The nuclear ataxin Q82 protein (Flag-Atx-Nuc) is detected with the Flag antibody. The HA antibody recognizes the CHPKs, and tubulin serves as a loading control. Numbers represent the different human herpesvirus CHPKs. EV, empty vector. (B) Lysates from transfected U-2 OS cells shown in Figure 6C were analyzed by Western blot with the indicated antibodies. The cytoplasmic ataxin Q82 K722T protein (Flag-Atx-Cyto) is detected with the Flag antibody. The HA antibody recognizes the CHPKs, and tubulin serves as a loading control. Numbers represent the different human herpesvirus CHPKs. EV, empty vector.
(0.41 MB TIF)

## References

[1] Alberts B (2002). Molecular biology of the cell.

[2] Arslan MA, Kutuk O, Basaga H (2006). Protein kinases as drug targets in cancer.. Curr Cancer Drug Targets.

[3] Trofe J, Pote L, Wade E, Blumberg E, Bloom RD (2008). Maribavir: a novel antiviral agent with activity against cytomegalovirus.. Ann Pharmacother.

[4] Drew WL (1991). Clinical use of ganciclovir for cytomegalovirus infection and the development of drug resistance.. J Acquir Immune Defic Syndr.

[5] Roizman B, Knipe DM, Whitley RJ, David M, Knipe PMH (2007). Herpes Simplex Viruses.. Fields' Virology.

[6] Cohen JI, Straus SE, Arvin AM, David M, Knipe PMH (2007). Varicella-Zoster Virus Replication, Pathogenesis, and Management.. Fields' Virology.

[7] Mocarski ES, Shenk T, Pass RF, David M, Knipe PMH (2007). Cytomegaloviruses.. Fields' Virology.

[8] Yamanashi K, Mori Y, Pellett PE, David M, Knipe PMH (2007). Human Herpesviruses 6 and 7.. Fields' Virology.

[9] Ganem D, David M, Knipe PMH (2007). Kaposi's Sarcoma-associated Herpesvirus.. Fields' Virology.

[10] Kieff ED, Rickinson AB, David M, Knipe PMH (2007). Epstein-Barr Virus and Its Replication.. Fields' Virology.

[11] Wiebe MS, Traktman P (2007). Poxviral B1 kinase overcomes barrier to autointegration factor, a host defense against virus replication.. Cell Host Microbe.

[12] Punjabi A, Traktman P (2005). Cell biological and functional characterization of the vaccinia virus F10 kinase: implications for the mechanism of virion morphogenesis.. J Virol.

[13] Gershburg E, Pagano JS (2008). Conserved herpesvirus protein kinases.. Biochim Biophys Acta.

[14] McGeoch DJ, Davison AJ (1986). Alphaherpesviruses possess a gene homologous to the protein kinase gene family of eukaryotes and retroviruses.. Nucleic Acids Res.

[15] Griffin AM, Boursnell ME (1990). Analysis of the nucleotide sequence of DNA from the region of the thymidine kinase gene of infectious laryngotracheitis virus; potential evolutionary relationships between the herpesvirus subfamilies.. J Gen Virol.

[16] Vende P, Taraporewala ZF, Patton JT (2002). RNA-binding activity of the rotavirus phosphoprotein NSP5 includes affinity for double-stranded RNA.. J Virol.

[17] Chung TD, Wymer JP, Smith CC, Kulka M, Aurelian L (1989). Protein kinase activity associated with the large subunit of herpes simplex virus type 2 ribonucleotide reductase (ICP10).. J Virol.

[18] Britt WJ, Auger D (1986). Human cytomegalovirus virion-associated protein with kinase activity.. J Virol.

[19] Benetti L, Munger J, Roizman B (2003). The herpes simplex virus 1 US3 protein kinase blocks caspase-dependent double cleavage and activation of the proapoptotic protein BAD.. J Virol.

[20] Ogg PD, McDonell PJ, Ryckman BJ, Knudson CM, Roller RJ (2004). The HSV-1 Us3 protein kinase is sufficient to block apoptosis induced by overexpression of a variety of Bcl-2 family members.. Virology.

[21] Morris JB, Hofemeister H, O'Hare P (2007). Herpes simplex virus infection induces phosphorylation and delocalization of emerin, a key inner nuclear membrane protein.. J Virol.

[22] Leach N, Bjerke SL, Christensen DK, Bouchard JM, Mou F (2007). Emerin is hyperphosphorylated and redistributed in herpes simplex virus type 1-infected cells in a manner dependent on both UL34 and US3.. J Virol.

[23] Mou F, Forest T, Baines JD (2007). US3 of herpes simplex virus type 1 encodes a promiscuous protein kinase that phosphorylates and alters localization of lamin A/C in infected cells.. J Virol.

[24] Shugar D (1999). Viral and host-cell protein kinases: enticing antiviral targets and relevance of nucleoside, and viral thymidine, kinases.. Pharmacol Ther.

[25] Smith RF, Smith TF (1989). Identification of new protein kinase-related genes in three herpesviruses, herpes simplex virus, varicella-zoster virus, and Epstein-Barr virus.. J Virol.

[26] Chee MS, Lawrence GL, Barrell BG (1989). Alpha-, beta- and gammaherpesviruses encode a putative phosphotransferase.. J Gen Virol.

[27] Kawaguchi Y, Kato K (2003). Protein kinases conserved in herpesviruses potentially share a function mimicking the cellular protein kinase cdc2.. Rev Med Virol.

[28] Purves FC, Ogle WO, Roizman B (1993). Processing of the herpes simplex virus regulatory protein alpha 22 mediated by the UL13 protein kinase determines the accumulation of a subset of alpha and gamma mRNAs and proteins in infected cells.. Proc Natl Acad Sci U S A.

[29] Heineman TC, Cohen JI (1995). The varicella-zoster virus (VZV) open reading frame 47 (ORF47) protein kinase is dispensable for viral replication and is not required for phosphorylation of ORF63 protein, the VZV homolog of herpes simplex virus ICP22.. J Virol.

[30] Moffat JF, Zerboni L, Sommer MH, Heineman TC, Cohen JI (1998). The ORF47 and ORF66 putative protein kinases of varicella-zoster virus determine tropism for human T cells and skin in the SCID-hu mouse.. Proc Natl Acad Sci U S A.

[31] Gershburg E, Raffa S, Torrisi MR, Pagano JS (2007). Epstein-Barr virus-encoded protein kinase (BGLF4) is involved in production of infectious virus.. J Virol.

[32] Prichard MN, Gao N, Jairath S, Mulamba G, Krosky P (1999). A recombinant human cytomegalovirus with a large deletion in UL97 has a severe replication deficiency.. J Virol.

[33] Tanaka M, Nishiyama Y, Sata T, Kawaguchi Y (2005). The role of protein kinase activity expressed by the UL13 gene of herpes simplex virus 1: the activity is not essential for optimal expression of UL41 and ICP0.. Virology.

[34] Izumiya Y, Izumiya C, Van Geelen A, Wang DH, Lam KS (2007). Kaposi's sarcoma-associated herpesvirus-encoded protein kinase and its interaction with K-bZIP.. J Virol.

[35] Stevenson D, Colman KL, Davison AJ (1994). Characterization of the putative protein kinases specified by varicella-zoster virus genes 47 and 66.. J Gen Virol.

[36] Wang JT, Yang PW, Lee CP, Han CH, Tsai CH (2005). Detection of Epstein-Barr virus BGLF4 protein kinase in virus replication compartments and virus particles.. J Gen Virol.

[37] van Zeijl M, Fairhurst J, Baum EZ, Sun L, Jones TR (1997). The human cytomegalovirus UL97 protein is phosphorylated and a component of virions.. Virology.

[38] Asai R, Kato A, Kato K, Kanamori-Koyama M, Sugimoto K (2006). Epstein-Barr virus protein kinase BGLF4 is a virion tegument protein that dissociates from virions in a phosphorylation-dependent process and phosphorylates the viral immediate-early protein BZLF1.. J Virol.

[39] Morrison EE, Wang YF, Meredith DM (1998). Phosphorylation of structural components promotes dissociation of the herpes simplex virus type 1 tegument.. J Virol.

[40] Long MC, Leong V, Schaffer PA, Spencer CA, Rice SA (1999). ICP22 and the UL13 protein kinase are both required for herpes simplex virus-induced modification of the large subunit of RNA polymerase II.. J Virol.

[41] Krosky PM, Baek MC, Jahng WJ, Barrera I, Harvey RJ (2003). The human cytomegalovirus UL44 protein is a substrate for the UL97 protein kinase.. J Virol.

[42] Marschall M, Freitag M, Suchy P, Romaker D, Kupfer R (2003). The protein kinase pUL97 of human cytomegalovirus interacts with and phosphorylates the DNA polymerase processivity factor pUL44.. Virology.

[43] Wolf DG, Courcelle CT, Prichard MN, Mocarski ES (2001). Distinct and separate roles for herpesvirus-conserved UL97 kinase in cytomegalovirus DNA synthesis and encapsidation.. Proc Natl Acad Sci U S A.

[44] Krosky PM, Baek MC, Coen DM (2003). The human cytomegalovirus UL97 protein kinase, an antiviral drug target, is required at the stage of nuclear egress.. J Virol.

[45] Hamirally S, Kamil JP, Ndassa-Colday YM, Lin AJ, Jahng WJ (2009). Viral mimicry of Cdc2/cyclin-dependent kinase 1 mediates disruption of nuclear lamina during human cytomegalovirus nuclear egress.. PLoS Pathog.

[46] Hume AJ, Finkel JS, Kamil JP, Coen DM, Culbertson MR (2008). Phosphorylation of retinoblastoma protein by viral protein with cyclin-dependent kinase function.. Science.

[47] Prichard MN, Sztul E, Daily SL, Perry AL, Frederick SL (2008). Human cytomegalovirus UL97 kinase activity is required for the hyperphosphorylation of retinoblastoma protein and inhibits the formation of nuclear aggresomes.. J Virol.

[48] Cano-Monreal GL, Tavis JE, Morrison LA (2008). Substrate specificity of the herpes simplex virus type 2 UL13 protein kinase.. Virology.

[49] Kawaguchi Y, Kato K, Tanaka M, Kanamori M, Nishiyama Y (2003). Conserved protein kinases encoded by herpesviruses and cellular protein kinase cdc2 target the same phosphorylation site in eukaryotic elongation factor 1delta.. J Virol.

[50] Kenyon TK, Lynch J, Hay J, Ruyechan W, Grose C (2001). Varicella-zoster virus ORF47 protein serine kinase: characterization of a cloned, biologically active phosphotransferase and two viral substrates, ORF62 and ORF63.. J Virol.

[51] Iwahori S, Murata T, Kudoh A, Sato Y, Nakayama S (2009). Phosphorylation of p27Kip1 by Epstein-Barr virus protein kinase induces its degradation through SCFSkp2 ubiquitin ligase actions during viral lytic replication.. J Biol Chem.

[52] Kamil JP, Coen DM (2007). Human cytomegalovirus protein kinase UL97 forms a complex with the tegument phosphoprotein pp65.. J Virol.

[53] Prichard MN, Britt WJ, Daily SL, Hartline CB, Kern ER (2005). Human cytomegalovirus UL97 Kinase is required for the normal intranuclear distribution of pp65 and virion morphogenesis.. J Virol.

[54] Ansari A, Emery VC (1999). The U69 gene of human herpesvirus 6 encodes a protein kinase which can confer ganciclovir sensitivity to baculoviruses.. J Virol.

[55] Hamza MS, Reyes RA, Izumiya Y, Wisdom R, Kung HJ (2004). ORF36 protein kinase of Kaposi's sarcoma herpesvirus activates the c-Jun N-terminal kinase signaling pathway.. J Biol Chem.

[56] Meng Q, Hagemeier SR, Kuny CV, Kalejta RF, Kenney SC Simian virus 40 T/t antigens and lamin A/C small interfering RNA rescue the phenotype of an Epstein-Barr virus protein kinase (BGLF4) mutant.. J Virol.

[57] Ng TI, Ogle WO, Roizman B (1998). UL13 protein kinase of herpes simplex virus 1 complexes with glycoprotein E and mediates the phosphorylation of the viral Fc receptor: glycoproteins E and I.. Virology.

[58] Daikoku T, Shibata S, Goshima F, Oshima S, Tsurumi T (1997). Purification and characterization of the protein kinase encoded by the UL13 gene of herpes simplex virus type 2.. Virology.

[59] Gershburg E, Marschall M, Hong K, Pagano JS (2004). Expression and localization of the Epstein-Barr virus-encoded protein kinase.. J Virol.

[60] Michel D, Pavic I, Zimmermann A, Haupt E, Wunderlich K (1996). The UL97 gene product of human cytomegalovirus is an early-late protein with a nuclear localization but is not a nucleoside kinase.. J Virol.

[61] De Bolle L, Michel D, Mertens T, Manichanh C, Agut H (2002). Role of the human herpesvirus 6 u69-encoded kinase in the phosphorylation of ganciclovir.. Mol Pharmacol.

[62] Isegawa Y, Miyamoto Y, Yasuda Y, Semi K, Tsujimura K (2008). Characterization of the human herpesvirus 6 U69 gene product and identification of its nuclear localization signal.. J Virol.

[63] Salsman J, Zimmerman N, Chen T, Domagala M, Frappier L (2008). Genome-wide screen of three herpesviruses for protein subcellular localization and alteration of PML nuclear bodies.. PLoS Pathog.

[64] Weinberg RA (1995). The retinoblastoma protein and cell cycle control.. Cell.

[65] Helt AM, Galloway DA (2003). Mechanisms by which DNA tumor virus oncoproteins target the Rb family of pocket proteins.. Carcinogenesis.

[66] Hume AJ, Kalejta RF (2009). Regulation of the retinoblastoma proteins by the human herpesviruses.. Cell Div.

[67] Kamil JP, Hume AJ, Jurak I, Munger K, Kalejta RF (2009). Human papillomavirus 16 E7 inactivator of retinoblastoma family proteins complements human cytomegalovirus lacking UL97 protein kinase.. Proc Natl Acad Sci U S A.

[68] Hinds PW, Mittnacht S, Dulic V, Arnold A, Reed SI (1992). Regulation of retinoblastoma protein functions by ectopic expression of human cyclins.. Cell.

[69] Chang Y, Moore PS, Talbot SJ, Boshoff CH, Zarkowska T (1996). Cyclin encoded by KS herpesvirus.. Nature.

[70] Knudsen ES, Wang JY (1996). Differential regulation of retinoblastoma protein function by specific Cdk phosphorylation sites.. J Biol Chem.

[71] Knudsen ES, Wang JY (1997). Dual mechanisms for the inhibition of E2F binding to RB by cyclin-dependent kinase-mediated RB phosphorylation.. Mol Cell Biol.

[72] Lee CP, Huang YH, Lin SF, Chang Y, Chang YH (2008). Epstein-Barr virus BGLF4 kinase induces disassembly of the nuclear lamina to facilitate virion production.. J Virol.

[73] Dechat T, Pfleghaar K, Sengupta K, Shimi T, Shumaker DK (2008). Nuclear lamins: major factors in the structural organization and function of the nucleus and chromatin.. Genes Dev.

[74] Mettenleiter TC (2002). Herpesvirus assembly and egress.. J Virol.

[75] Heald R, McKeon F (1990). Mutations of phosphorylation sites in lamin A that prevent nuclear lamina disassembly in mitosis.. Cell.

[76] Cano-Monreal GL, Wylie KM, Cao F, Tavis JE, Morrison LA (2009). Herpes simplex virus 2 UL13 protein kinase disrupts nuclear lamins.. Virology.

[77] Lee MG, Nurse P (1987). Complementation used to clone a human homologue of the fission yeast cell cycle control gene cdc2.. Nature.

[78] Reed SI, Wittenberg C (1990). Mitotic role for the Cdc28 protein kinase of Saccharomyces cerevisiae.. Proc Natl Acad Sci U S A.

[79] Ninomiya-Tsuji J, Nomoto S, Yasuda H, Reed SI, Matsumoto K (1991). Cloning of a human cDNA encoding a CDC2-related kinase by complementation of a budding yeast cdc28 mutation.. Proc Natl Acad Sci U S A.

[80] Verschuren EW, Jones N, Evan GI (2004). The cell cycle and how it is steered by Kaposi's sarcoma-associated herpesvirus cyclin.. J Gen Virol.

[81] Bernardi R, Pandolfi PP (2007). Structure, dynamics and functions of promyelocytic leukaemia nuclear bodies.. Nat Rev Mol Cell Biol.

[82] Everett RD, Murray J (2005). ND10 components relocate to sites associated with herpes simplex virus type 1 nucleoprotein complexes during virus infection.. J Virol.

[83] Ishov AM, Stenberg RM, Maul GG (1997). Human cytomegalovirus immediate early interaction with host nuclear structures: definition of an immediate transcript environment.. J Cell Biol.

[84] Saffert RT, Kalejta RF (2008). Promyelocytic leukemia-nuclear body proteins: herpesvirus enemies, accomplices, or both?. Future Virology.

[85] Tavalai N, Stamminger T (2008). New insights into the role of the subnuclear structure ND10 for viral infection.. Biochim Biophys Acta.

[86] Korioth F, Maul GG, Plachter B, Stamminger T, Frey J (1996). The nuclear domain 10 (ND10) is disrupted by the human cytomegalovirus gene product IE1.. Exp Cell Res.

[87] Saffert RT, Kalejta RF (2006). Inactivating a cellular intrinsic immune defense mediated by Daxx is the mechanism through which the human cytomegalovirus pp71 protein stimulates viral immediate-early gene expression.. J Virol.

[88] Maul GG, Guldner HH, Spivack JG (1993). Modification of discrete nuclear domains induced by herpes simplex virus type 1 immediate early gene 1 product (ICP0).. J Gen Virol.

[89] Livingston CM, Ifrim MF, Cowan AE, Weller SK (2009). Virus-Induced Chaperone-Enriched (VICE) domains function as nuclear protein quality control centers during HSV-1 infection.. PLoS Pathog.

[90] Klement IA, Skinner PJ, Kaytor MD, Yi H, Hersch SM (1998). Ataxin-1 nuclear localization and aggregation: role in polyglutamine-induced disease in SCA1 transgenic mice.. Cell.

[91] Baek MC, Krosky PM, Pearson A, Coen DM (2004). Phosphorylation of the RNA polymerase II carboxyl-terminal domain in human cytomegalovirus-infected cells and in vitro by the viral UL97 protein kinase.. Virology.

[92] Kudoh A, Daikoku T, Ishimi Y, Kawaguchi Y, Shirata N (2006). Phosphorylation of MCM4 at sites inactivating DNA helicase activity of the MCM4-MCM6-MCM7 complex during Epstein-Barr virus productive replication.. J Virol.

[93] Lee CP, Chen JY, Wang JT, Kimura K, Takemoto A (2007). Epstein-Barr virus BGLF4 kinase induces premature chromosome condensation through activation of condensin and topoisomerase II.. J Virol.

[94] Kudoh A, Fujita M, Kiyono T, Kuzushima K, Sugaya Y (2003). Reactivation of lytic replication from B cells latently infected with Epstein-Barr virus occurs with high S-phase cyclin-dependent kinase activity while inhibiting cellular DNA replication.. J Virol.

[95] Jault FM, Jault JM, Ruchti F, Fortunato EA, Clark C (1995). Cytomegalovirus infection induces high levels of cyclins, phosphorylated Rb, and p53, leading to cell cycle arrest.. J Virol.

[96] Marschall M, Marzi A, aus dem Siepen P, Jochmann R, Kalmer M (2005). Cellular p32 recruits cytomegalovirus kinase pUL97 to redistribute the nuclear lamina.. J Biol Chem.

[97] McLaughlin-Drubin ME, Munger K (2009). The human papillomavirus E7 oncoprotein.. Virology.

[98] Mou F, Wills EG, Park R, Baines JD (2008). Effects of lamin A/C, lamin B1, and viral US3 kinase activity on viral infectivity, virion egress, and the targeting of herpes simplex virus U(L)34-encoded protein to the inner nuclear membrane.. J Virol.

[99] Malumbres M, Barbacid M (2005). Mammalian cyclin-dependent kinases.. Trends Biochem Sci.

[100] Malumbres M, Harlow E, Hunt T, Hunter T, Lahti JM (2009). Cyclin-dependent kinases: a family portrait.. Nat Cell Biol.

[101] Ahn JH, Hayward GS (1997). The major immediate-early proteins IE1 and IE2 of human cytomegalovirus colocalize with and disrupt PML-associated nuclear bodies at very early times in infected permissive cells.. J Virol.

[102] Adamson AL, Kenney S (2001). Epstein-barr virus immediate-early protein BZLF1 is SUMO-1 modified and disrupts promyelocytic leukemia bodies.. J Virol.

[103] Wileman T (2007). Aggresomes and pericentriolar sites of virus assembly: cellular defense or viral design?. Annu Rev Microbiol.

[104] Burch AD, Weller SK (2004). Nuclear sequestration of cellular chaperone and proteasomal machinery during herpes simplex virus type 1 infection.. J Virol.

[105] Burch AD, Weller SK (2005). Herpes simplex virus type 1 DNA polymerase requires the mammalian chaperone hsp90 for proper localization to the nucleus.. J Virol.

[106] Nozawa N, Yamauchi Y, Ohtsuka K, Kawaguchi Y, Nishiyama Y (2004). Formation of aggresome-like structures in herpes simplex virus type 2-infected cells and a potential role in virus assembly.. Exp Cell Res.

[107] Ng TI, Talarico C, Burnette TC, Biron K, Roizman B (1996). Partial substitution of the functions of the herpes simplex virus 1 U(L)13 gene by the human cytomegalovirus U(L)97 gene.. Virology.

[108] Romaker D, Schregel V, Maurer K, Auerochs S, Marzi A (2006). Analysis of the structure-activity relationship of four herpesviral UL97 subfamily protein kinases reveals partial but not full functional conservation.. J Med Chem.

[109] Davison AJ, Ann Arvin GC-F, Mocarski Edward, Moore PatrickS, Roizman Bernard, Whitley Richard, Yamanishi Koichi (2007). Comparative Analysis of the Genomes.. Human Herpesviruses: Biology, Therapy, and Immunoprophylaxis.

[110] Pellet PE, Roizman B, David M, Knipe PMH (2007). The Family *Herpesviridae*: A Brief Introduction.. Fields' Virology.

[111] Miller ME, Engel DA, Smith MM (1995). Cyclic AMP signaling is required for function of the N-terminal and CR1 domains of adenovirus E1A in Saccharomyces cerevisiae.. Oncogene.

[112] Marschall M, Stein-Gerlach M, Freitag M, Kupfer R, van den Bogaard M (2002). Direct targeting of human cytomegalovirus protein kinase pUL97 by kinase inhibitors is a novel principle for antiviral therapy.. J Gen Virol.

[113] Neipel F, Ellinger K, Fleckenstein B (1991). The unique region of the human herpesvirus 6 genome is essentially collinear with the UL segment of human cytomegalovirus.. J Gen Virol.

[114] Tamura K, Dudley J, Nei M, Kumar S (2007). MEGA4: Molecular Evolutionary Genetics Analysis (MEGA) software version 4.0.. Mol Biol Evol.

[115] Kalejta RF, Bechtel JT, Shenk T (2003). Human cytomegalovirus pp71 stimulates cell cycle progression by inducing the proteasome-dependent degradation of the retinoblastoma family of tumor suppressors.. Mol Cell Biol.

[116] Saffert RT, Kalejta RF (2007). Human cytomegalovirus gene expression is silenced by Daxx-mediated intrinsic immune defense in model latent infections established in vitro.. J Virol.

[117] Zhu H, Shen Y, Shenk T (1995). Human cytomegalovirus IE1 and IE2 proteins block apoptosis.. J Virol.

[118] Hanks SK, Quinn AM, Hunter T (1988). The protein kinase family: conserved features and deduced phylogeny of the catalytic domains.. Science.

[119] Russo AA, Jeffrey PD, Patten AK, Massague J, Pavletich NP (1996). Crystal structure of the p27Kip1 cyclin-dependent-kinase inhibitor bound to the cyclin A-Cdk2 complex.. Nature.

[120] Russo AA, Tong L, Lee JO, Jeffrey PD, Pavletich NP (1998). Structural basis for inhibition of the cyclin-dependent kinase Cdk6 by the tumour suppressor p16INK4a.. Nature.

[121] Jeffrey PD, Russo AA, Polyak K, Gibbs E, Hurwitz J (1995). Mechanism of CDK activation revealed by the structure of a cyclinA-CDK2 complex.. Nature.

