# Supplementary figures and images for: Cyclin-Dependent Kinase-Like Function Is Shared by the Beta- and Gamma- Subset of the Conserved Herpesvirus Protein Kinases
Source: PLoS Pathog. 2010 Sep 9;6(9):e1001092. doi: 10.1371/journal.ppat.1001092 (PMC2936540; doi:10.1371/journal.ppat.1001092)

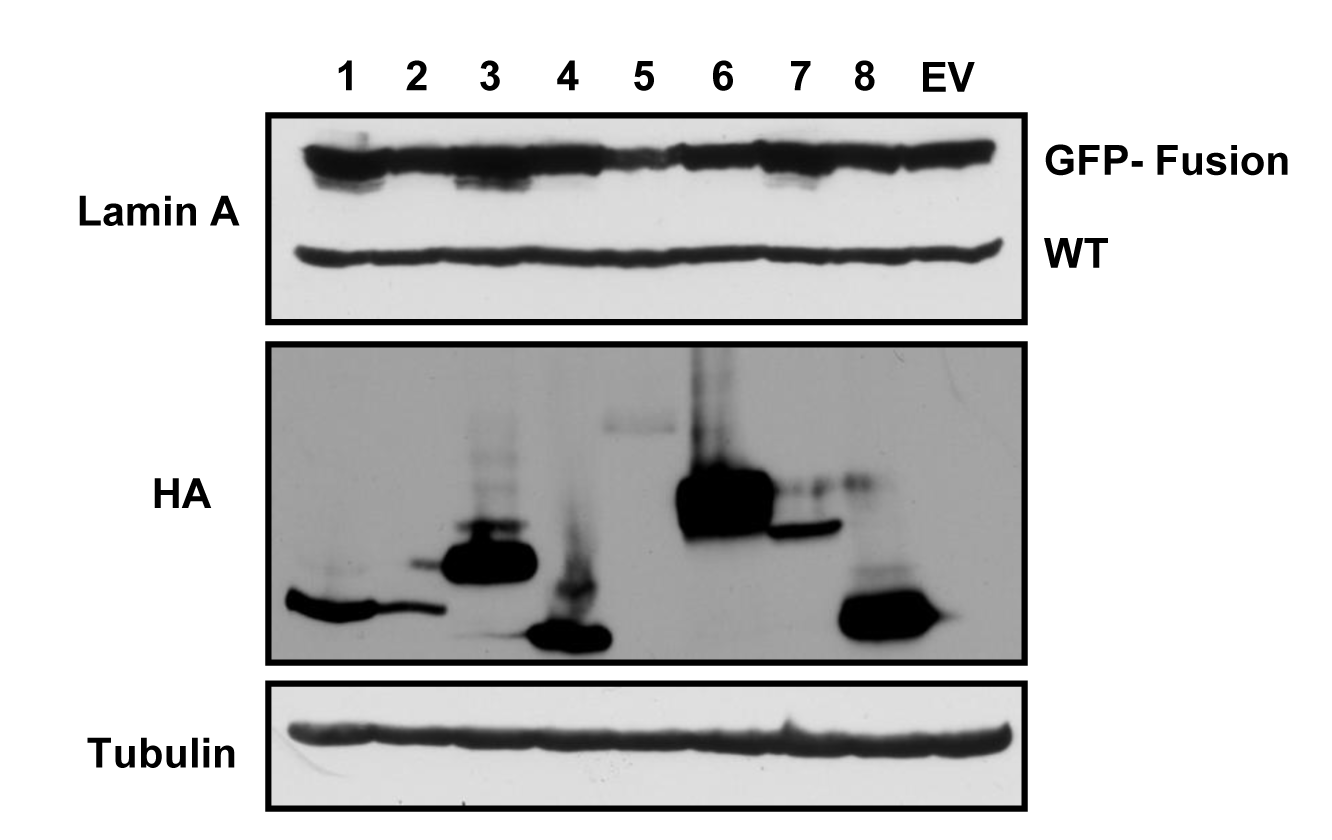

Supplement: Figure S1 — Western blot analysis of lysates from the lamina disruption experiment presented in Figure 3A. Lysates from the transfected U-2 OS cells shown in Figure 3A were analyzed by Western blot with the indicated antibodies. Note that these cells express both endogenous lamin A as well as the ectopic lamin A-GFP fusion protein, and both are recognized by the lamin A antibody. The HA antibody recognizes the CHPKs, and tubulin serves as a loading control. Numbers represent the different human herpesvirus CHPKs. EV, empty vector. (0.23 MB TIF) [file ppat.1001092.s001.tif]

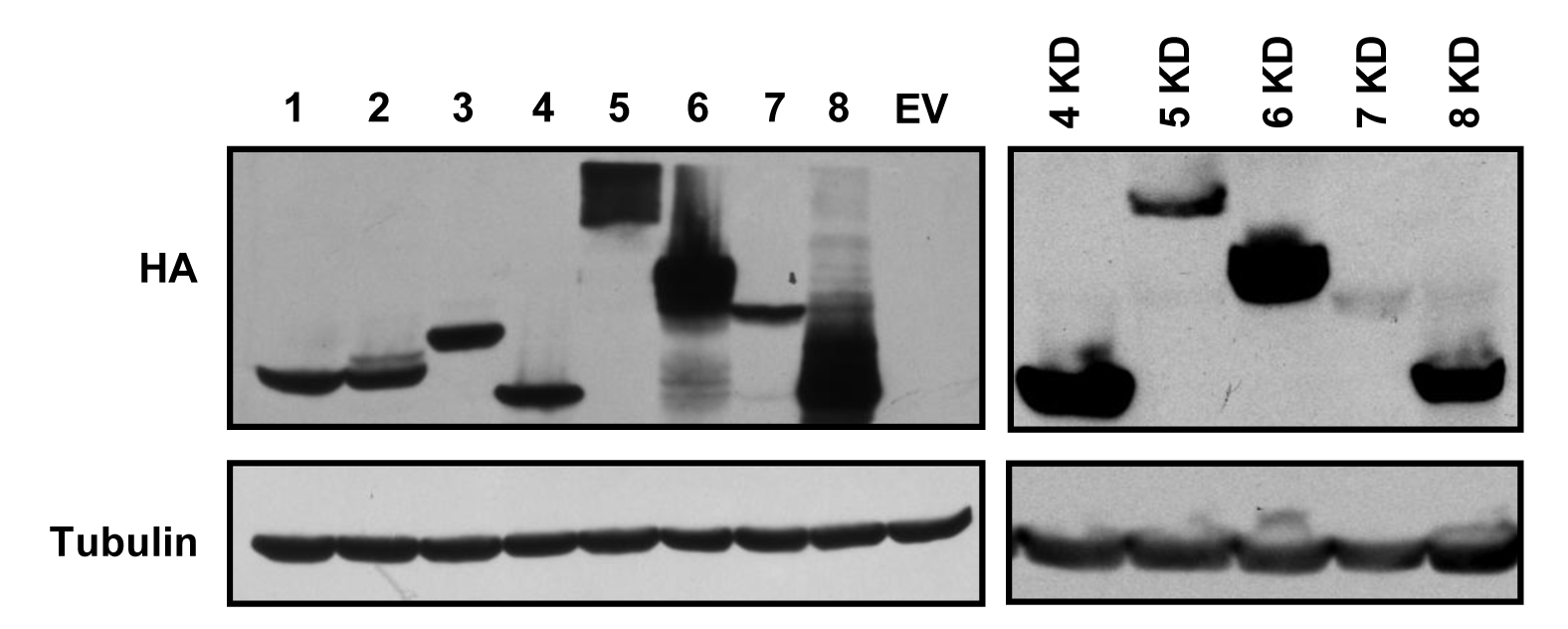

Supplement: Figure S2 — Western blot analysis of lysates from the lamin A phosphorylation experiment presented in Figures 3C, 3D, and 3E. Lysates from the transfected U-2 OS cells shown in Figure 3C, 3D, and 3E were analyzed by Western blot with the indicated antibodies. The HA antibody recognizes both wild type and kinase deficient (KD) CHPKs, and tubulin serves as a loading control. Numbers represent the different human herpesvirus CHPKs. EV, empty vector. (0.36 MB TIF) [file ppat.1001092.s002.tif]

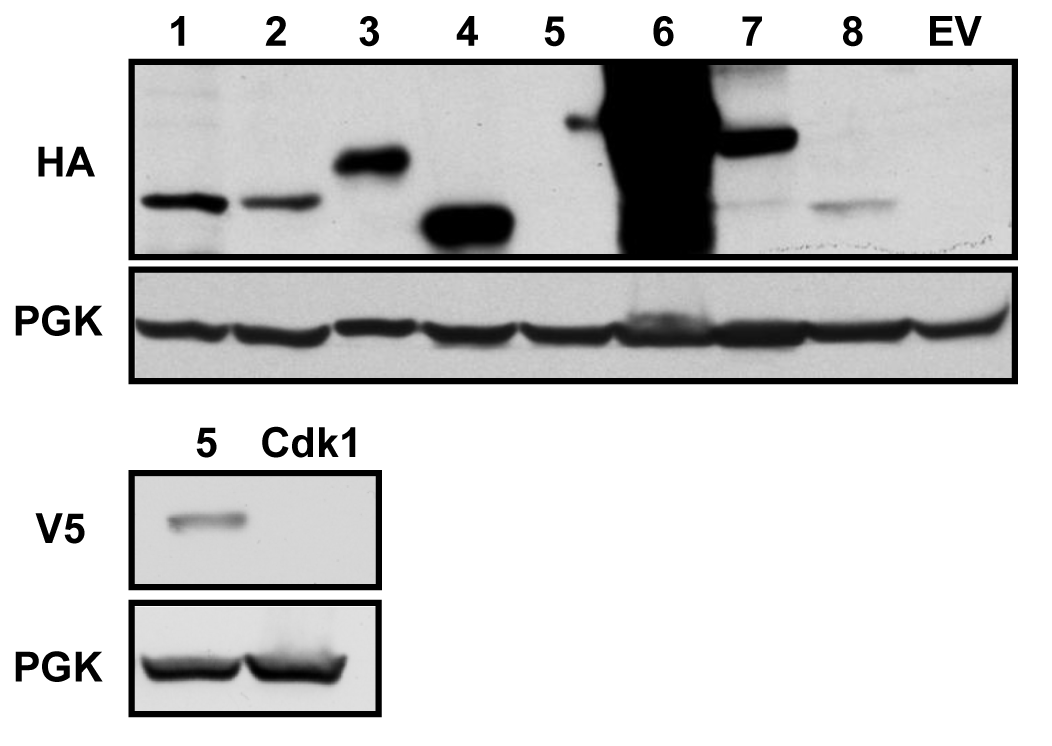

Supplement: Figure S3 — Western blot analysis of lysates from the S. cerevisiae Cdk complementation asay shown in Figure 4. Lysates from S. cerevisiae harboring plasmids expressing the indicated kinase and treated with galactose were analyzed by Western blot with the indicated antibodies. The HA antibody recognizes all CHPKs except for the HHV-5 protein, which is visualized with the V5 antibody. Cdk1 expression was not analyzed. PGK (3-Phosphoglycerokinase) serves as a loading control. Numbers represent the different human herpesvirus CHPKs. EV, empty vector. (0.22 MB TIF) [file ppat.1001092.s003.tif]

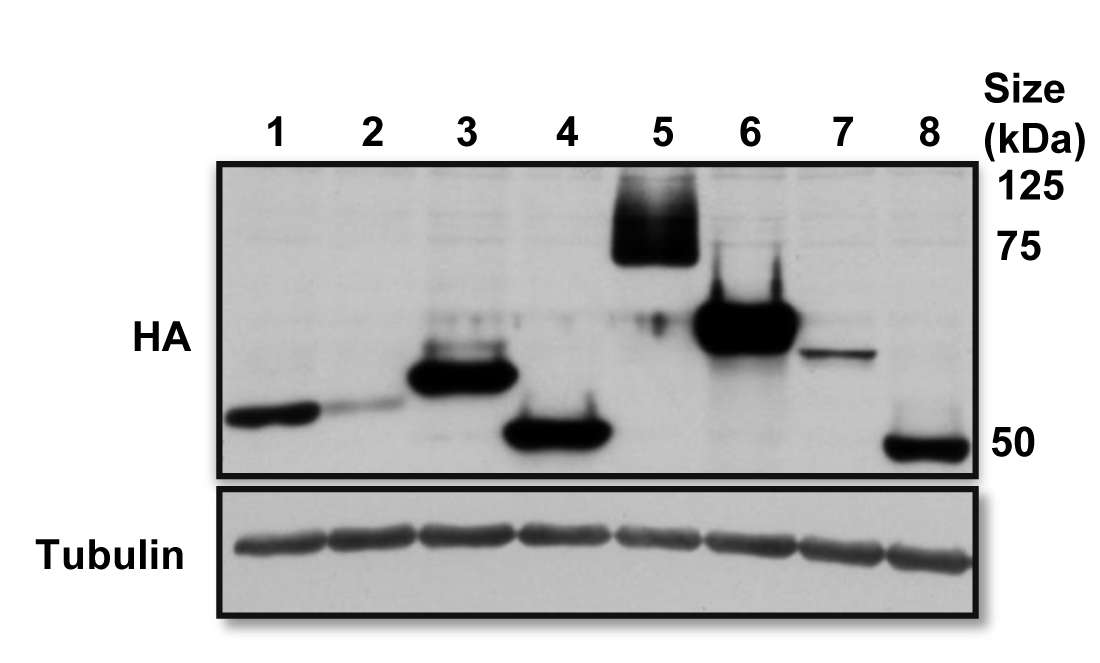

Supplement: Figure S4 — Western blot analysis of lysates from the PML-NB disruption experiment presented in Figure 5. Lysates from transfected U-2 OS cells shown in Figure 5A were analyzed by Western blot with the indicated antibodies. The HA antibody recognizes the CHPK, and tubulin serves as a loading control. The approximate localization of molecular weight markers (in Kilo Daltons, kDa) is displayed. Numbers represent the different human herpesvirus CHPKs. (0.20 MB TIF) [file ppat.1001092.s004.tif]

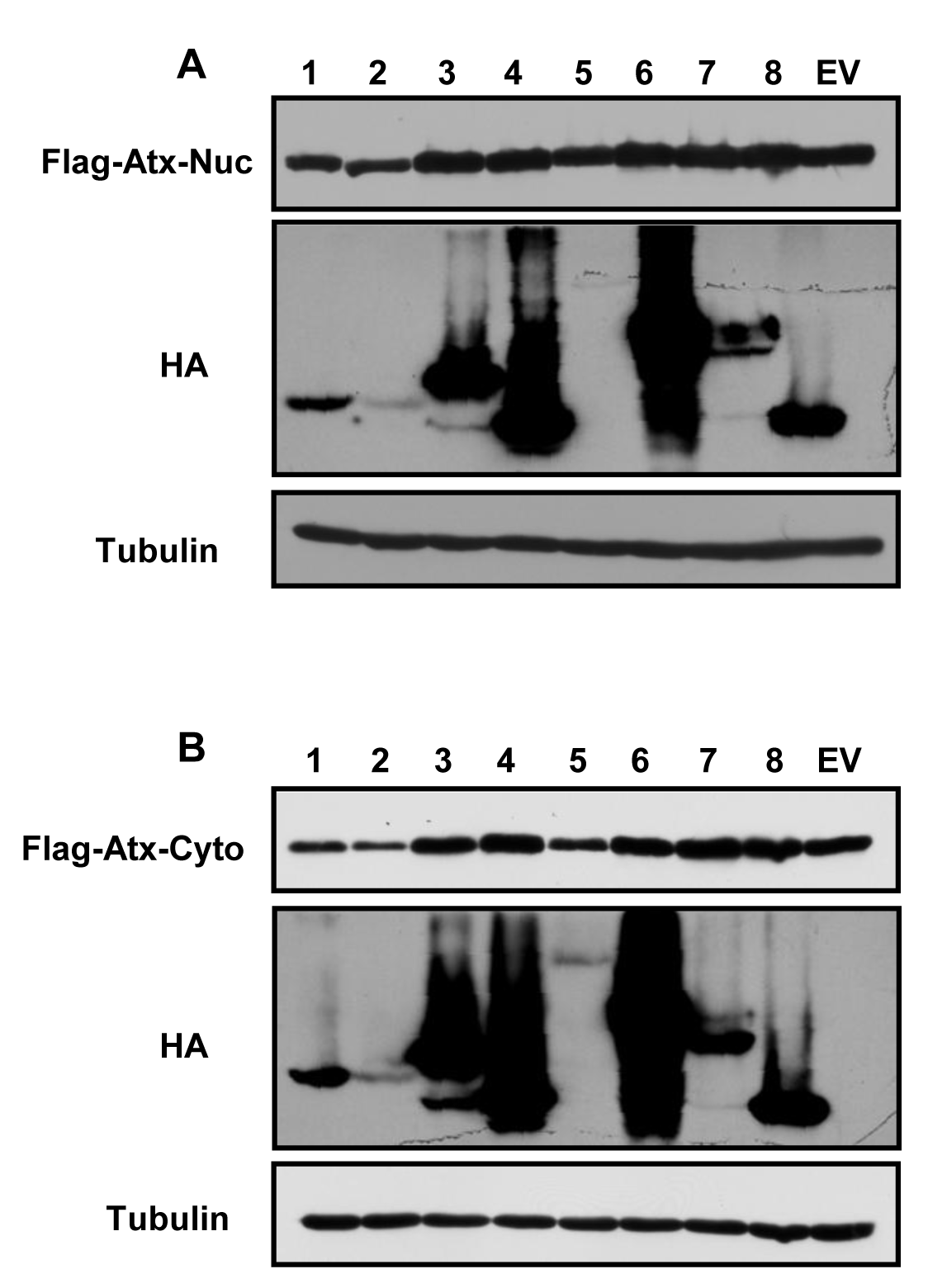

Supplement: Figure S5 — Western blot analysis of lysates from the aggresome disruption experiments presented in Figure 6. (A) Lysates from transfected U-2 OS cells shown in Figure 6A were analyzed by Western blot with the indicated antibodies. The nuclear ataxin Q82 protein (Flag-Atx-Nuc) is detected with the Flag antibody. The HA antibody recognizes the CHPKs, and tubulin serves as a loading control. Numbers represent the different human herpesvirus CHPKs. EV, empty vector. (B) Lysates from transfected U-2 OS cells shown in Figure 6C were analyzed by Western blot with the indicated antibodies. The cytoplasmic ataxin Q82 K722T protein (Flag-Atx-Cyto) is detected with the Flag antibody. The HA antibody recognizes the CHPKs, and tubulin serves as a loading control. Numbers represent the different human herpesvirus CHPKs. EV, empty vector. (0.41 MB TIF) [file ppat.1001092.s005.tif]
